# Supplementary figures and images for: Spatial information allows inference of the prevalence of direct cell–to–cell viral infection
Source: PLoS Comput Biol. 2024 Jul 23;20(7):e1012264. doi: 10.1371/journal.pcbi.1012264 (PMC11296656; doi:10.1371/journal.pcbi.1012264)

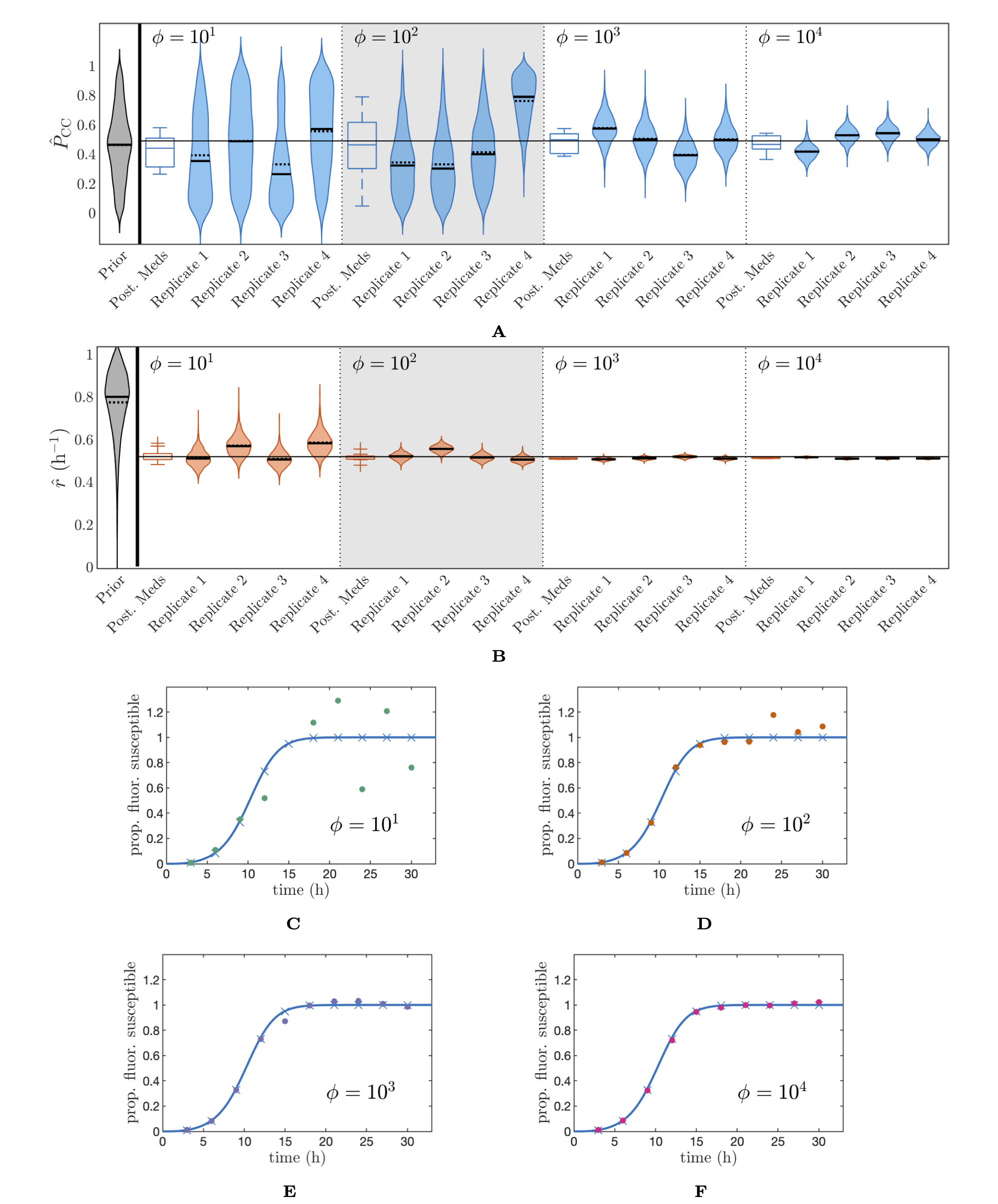

Supplement: S1 Fig — (A) Prior density and posterior densities from individual replicates for PCC at different levels of observational noise. At each level of noise we also show a box plot of the distribution of posterior medians across all replicates. There are ten replicates in total at each level of noise, of which we display four. The highlighted segment is the level of noise used in the main text. (B) Same as (A), but showing estimates for r. (C)–(F) Indicative observed data compared to true fluorescence time series for each value of the dispersion parameter ϕ used in (A) and (B). Here α = 1.09h-1, β = 7.20 × 10^−7^(TCID50/ml)-1h-1, with PCC ≈ 0.5. (TIFF) [file pcbi.1012264.s001.tiff]

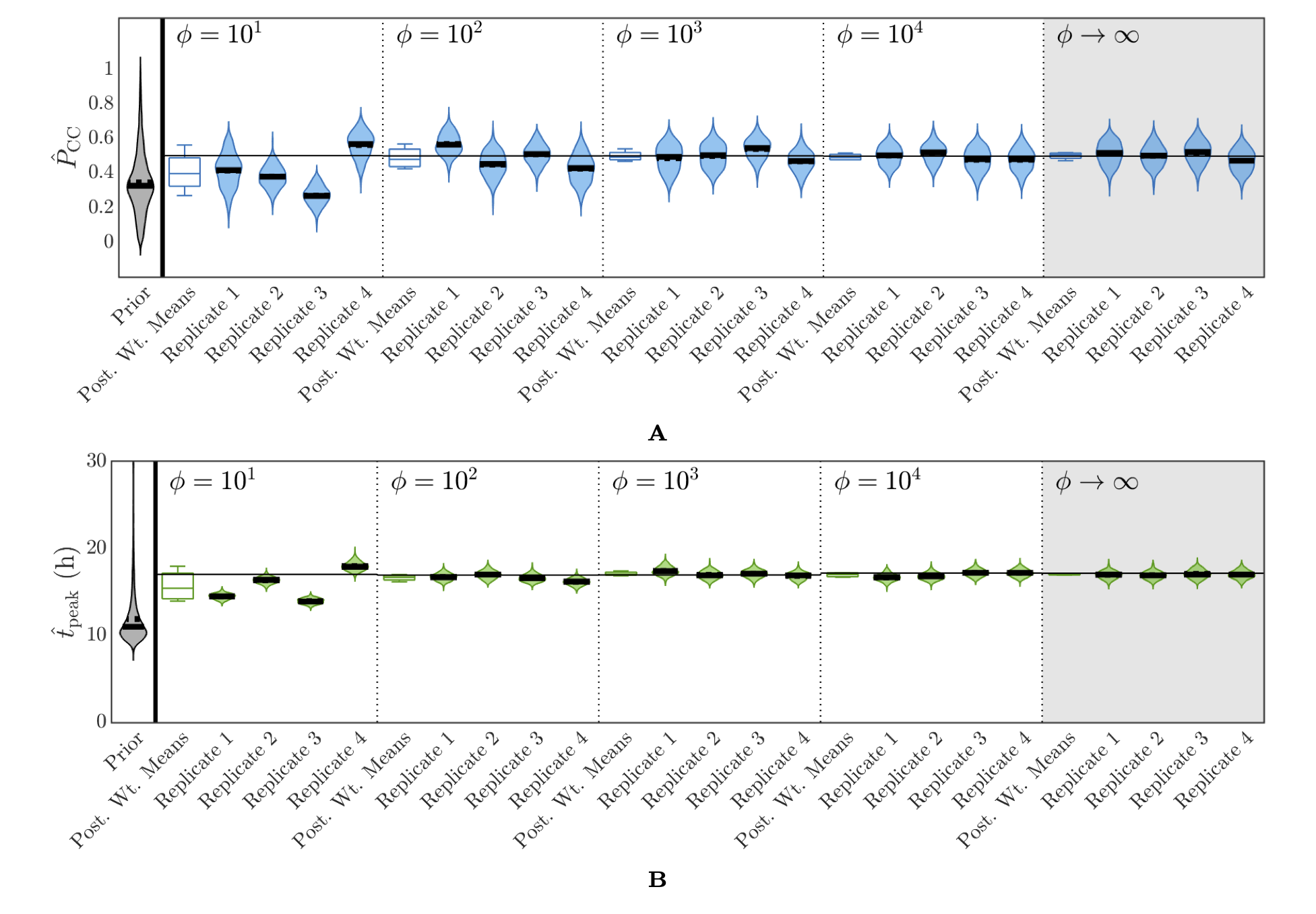

Supplement: S2 Fig — (A) Prior density and posterior densities from individual replicates for PCC at different levels of observational noise. At each level of noise we also show a box plot of the distribution of posterior medians across all replicates. There are four replicates at each level of noise. The highlighted segment is the level of noise used in the main text (which in this case has no artificial observational noise beyond the inherent stochasticity of the model, as explained in the main text). (B) Same as (A), but showing estimates for tpeak. Here α = 1.11h-1, β = 3.91 × 10^−7^(TCID50/ml)-1h-1, with PCC ≈ 0.5. (TIFF) [file pcbi.1012264.s002.tiff]

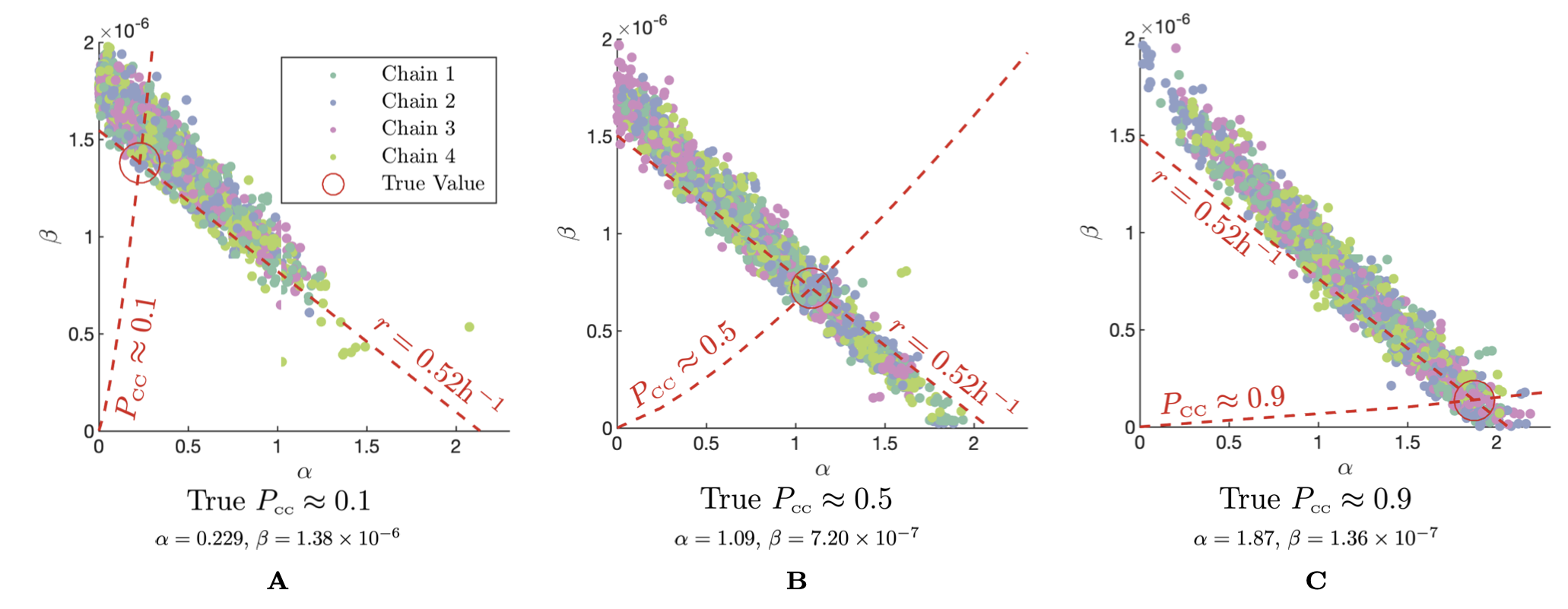

Supplement: S3 Fig — Scatter plot of accepted posterior samples in α–β space for a fit to fluorescence data where the true PCC ≈ 0.1, 0.5, 0.9 and fixed r using the ODE model, as presented in Fig 2 of the main article. (TIFF) [file pcbi.1012264.s003.tiff]

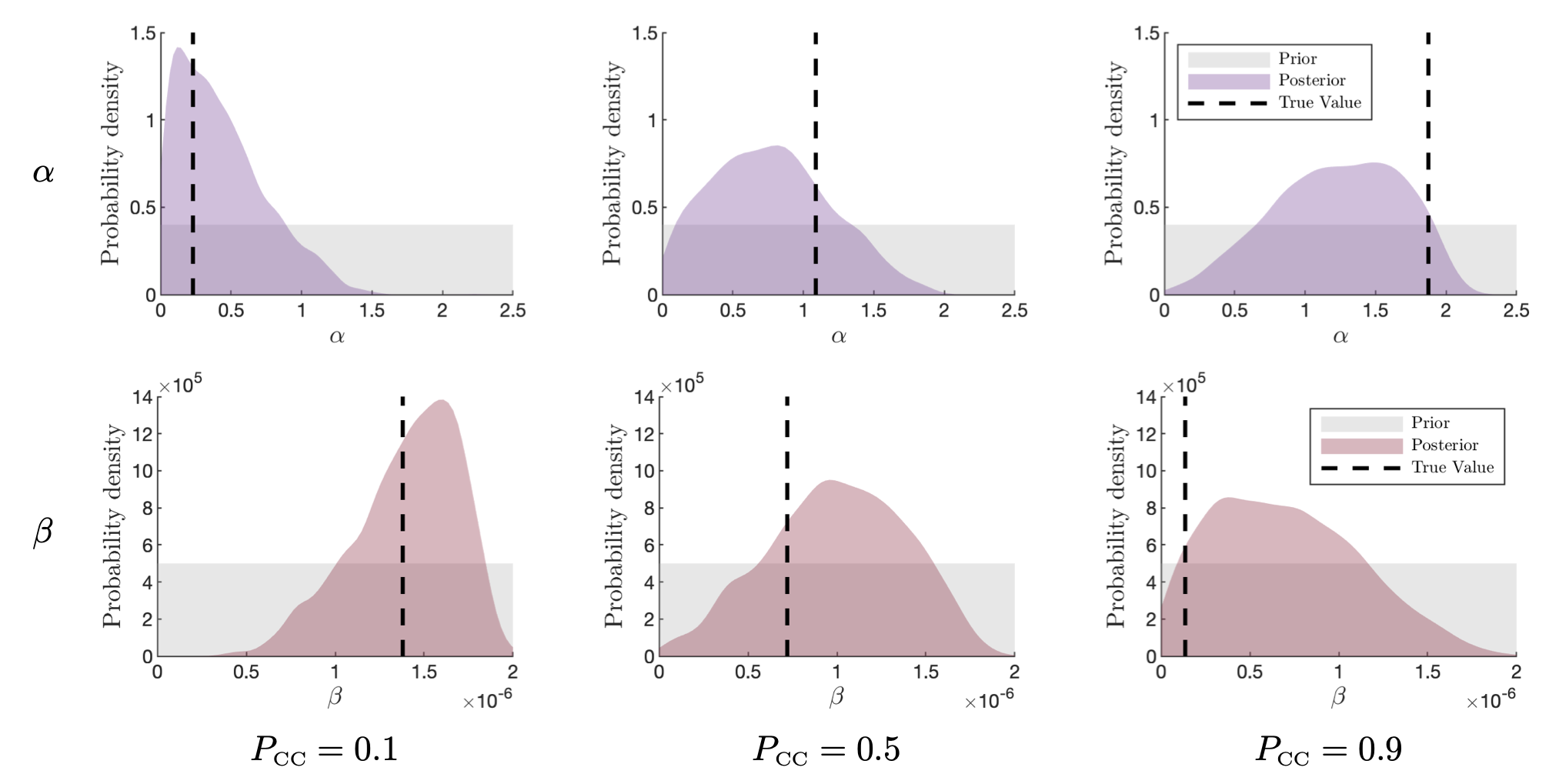

Supplement: S4 Fig — Posterior and prior distributions for α and β for simulation–estimations with the ODE model presented in Fig 2 of the main article. (TIFF) [file pcbi.1012264.s004.tiff]

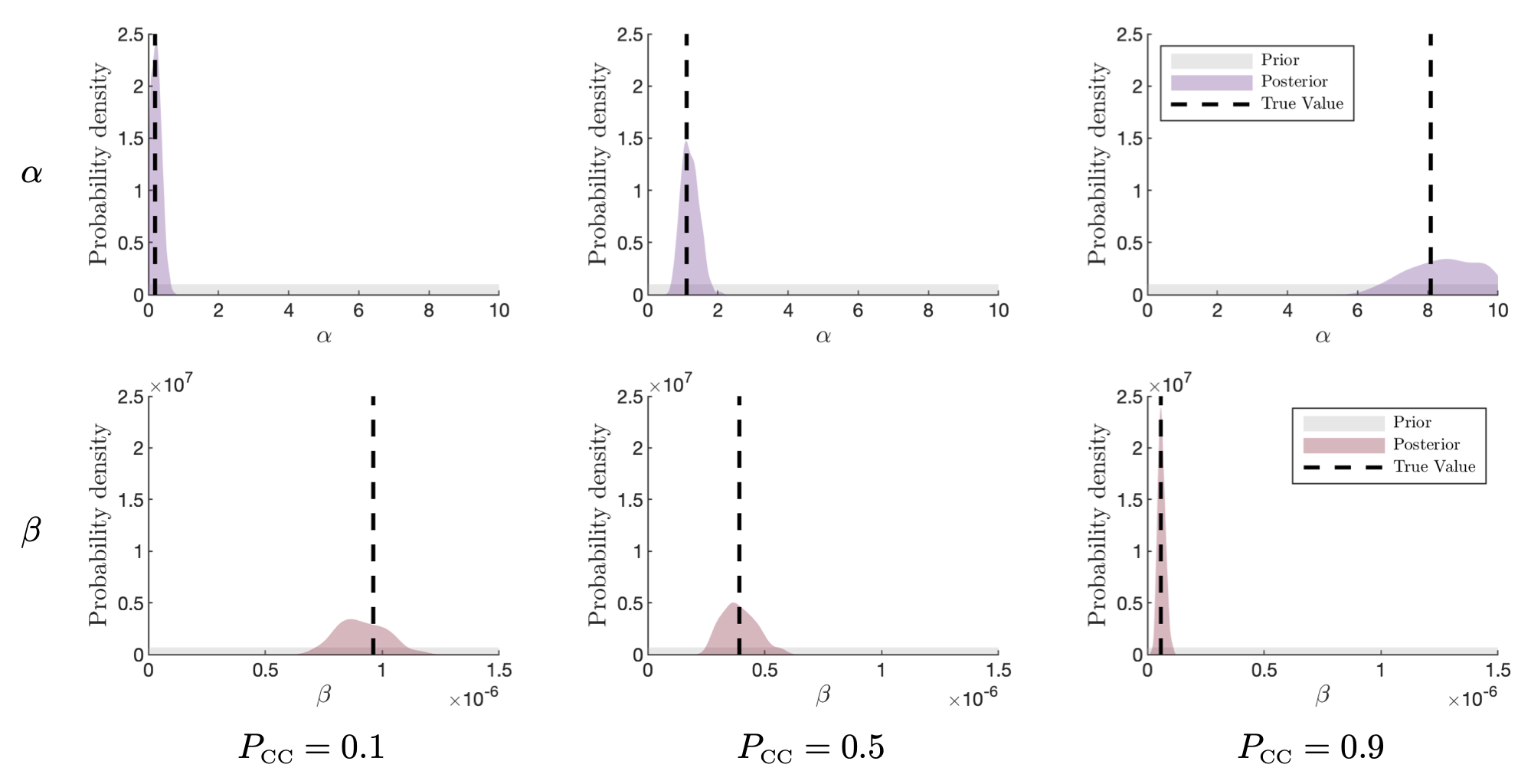

Supplement: S5 Fig — Posterior and prior distributions for α and β for simulation–estimations with the spatial model (with the clustering metric) presented in Fig 4 of the main article. (TIFF) [file pcbi.1012264.s005.tiff]

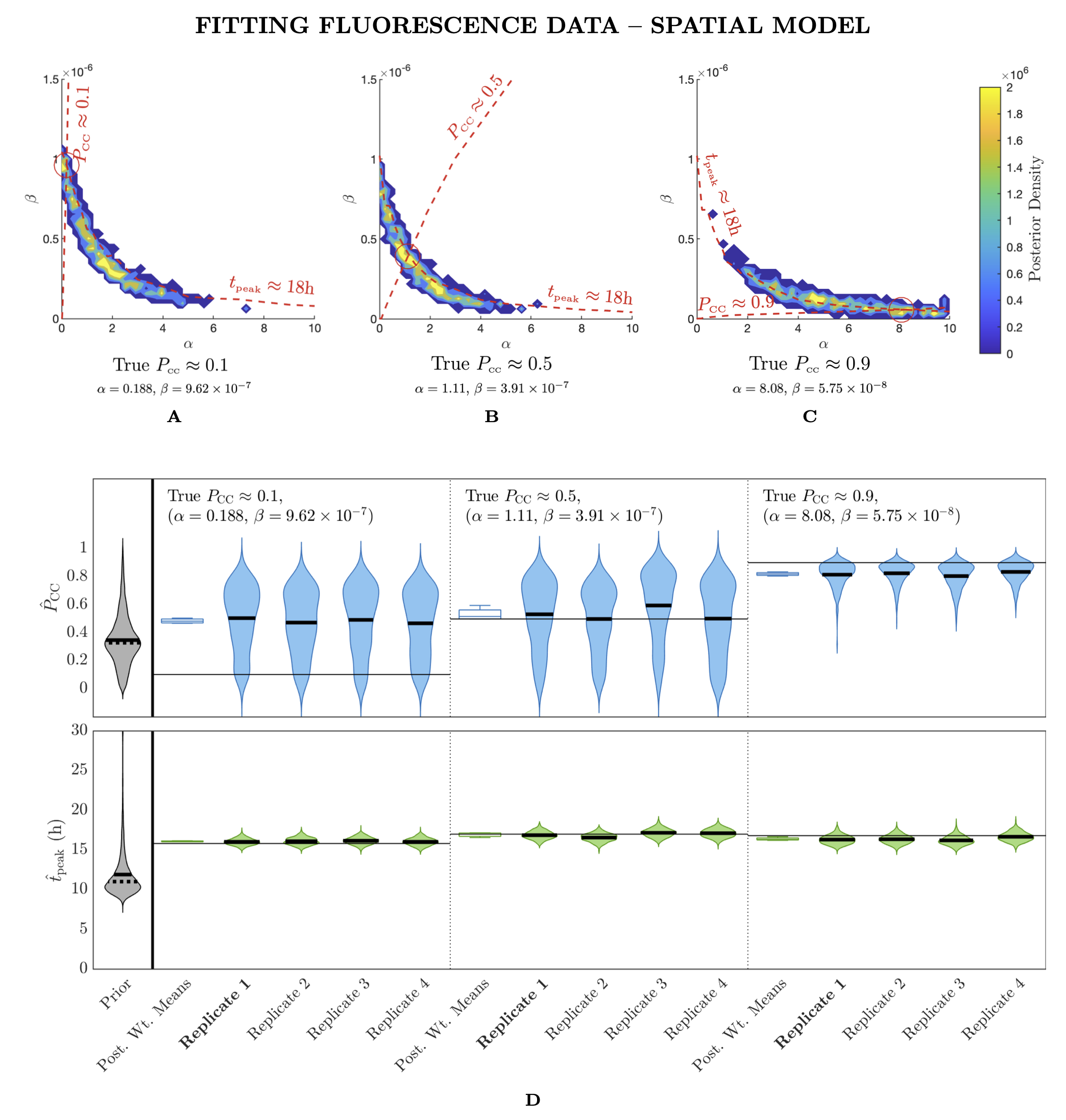

Supplement: S6 Fig — (A)–(C) Posterior density in α–β space for a fit to fluorescence data where the true PCC ≈ 0.1, 0.5, 0.9 and the infected cell peak time is held fixed at approximately 18h. We only show densities above a threshold value of 10−4. (D) Prior density and posterior densities from individual replicates for infected peak time and PCC with target parameters as specified in (A)–(C). Dashed and solid horizontal lines mark the weighted mean and median values respectively. We also show a box plot of the distribution of posterior weighted means across all four replicates in each case. The replicates in bold are those plotted in (A)–(C). α and β have units of h-1 and (TCID50/ml)-1h-1, respectively. (TIFF) [file pcbi.1012264.s006.tiff]

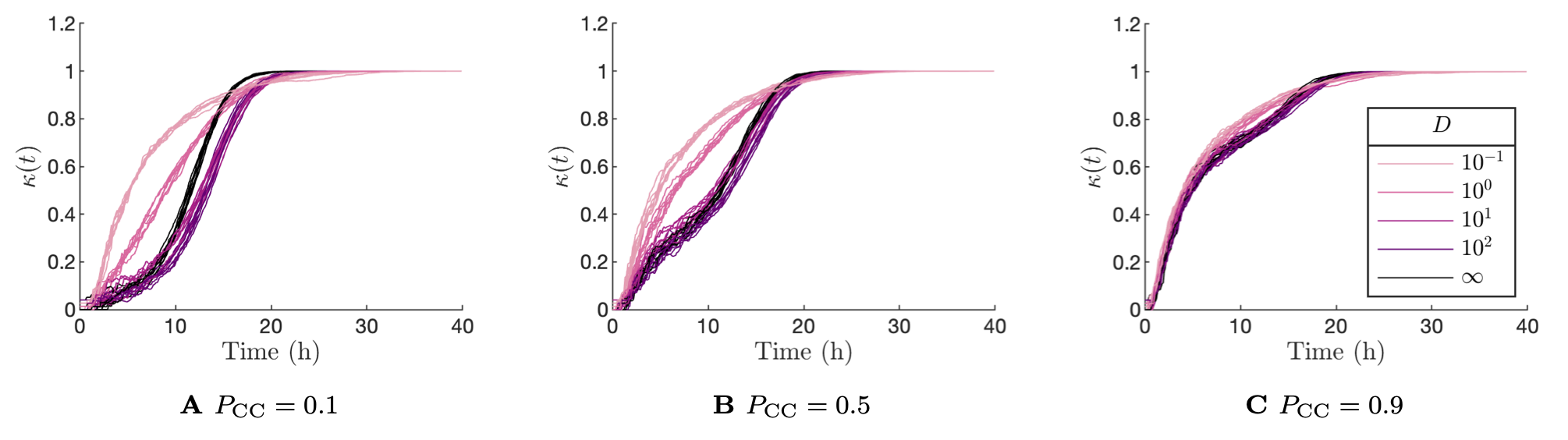

Supplement: S7 Fig — The clustering metric, κ(t) for the indicated values of the extracellular viral diffusion coefficient D, where and α and β are chosen such that PCC values are approximately 0.1, 0.5, and 0.9 and tpeak is approximately 18h for the specified value of D (according to Table A in S5 Text). We show results from eight simulations in each case. These are the same κ(t) trajectories as in Fig 5B–5F in the main text but grouped by PCC. Note that there is some noise associated with the parameter selections for finite diffusion since the lookup tables used are coarser than that for the infinite diffusion model, hence the curves shown only approximately correspond to the indicated PCC and tpeak values. (TIFF) [file pcbi.1012264.s007.tiff]

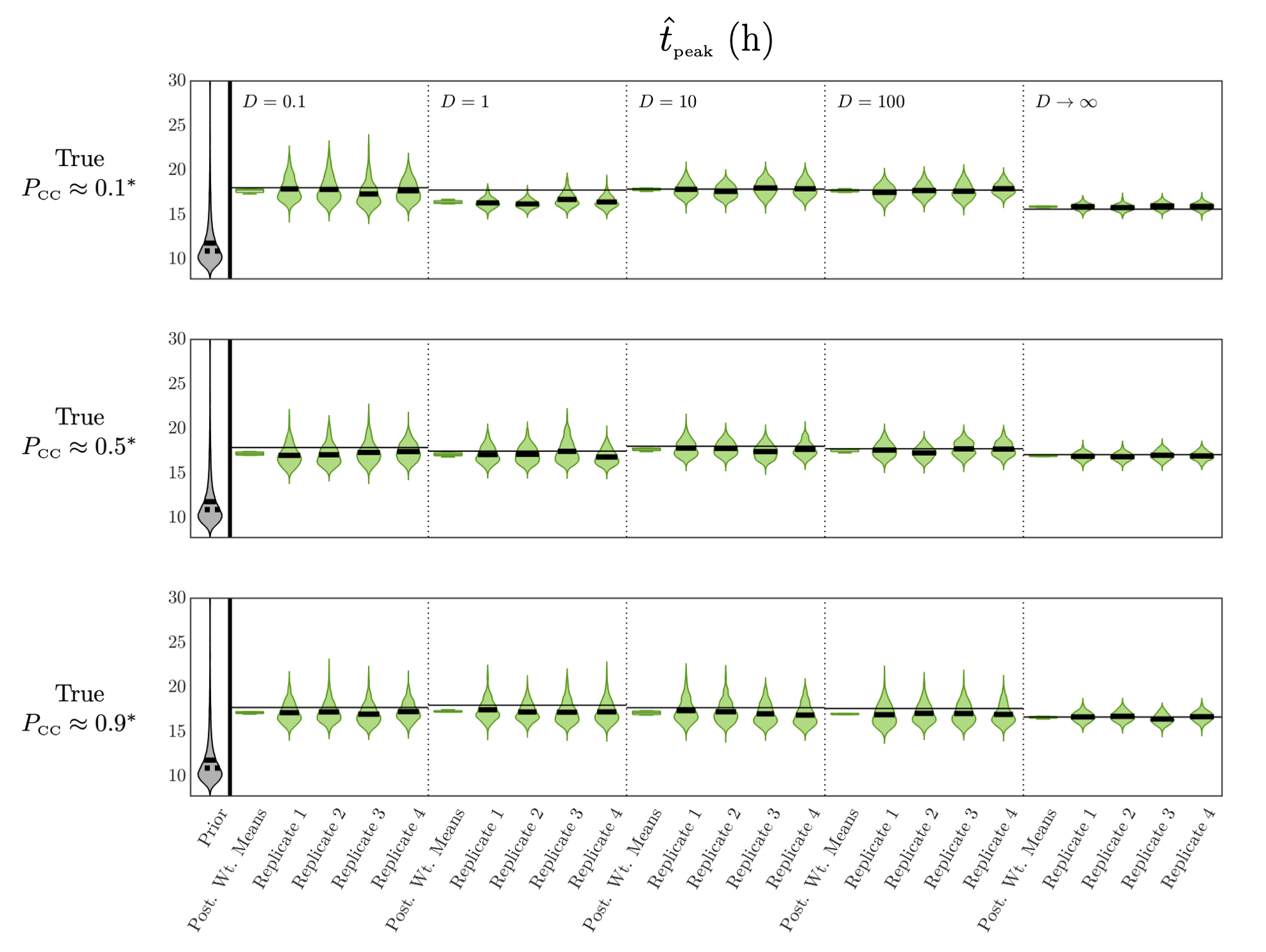

Supplement: S8 Fig — Prior density and posterior densities from individual replicates for tpeak for different values of D, the value of the extracellular viral diffusion coefficient used in the extended spatial model to generate observational data. We re–fit using the basic spatial model. For each value of D we also show a boxplot of the distribution of posterior weighted means across all four replicates. We show results for the case where the target values of α and β give rise to PCC values of approximately 0.1, 0.5, and 0.9 and tpeak of approximately 18h for the specified value of D. α and β values for each D values used are specified in Table A in S6 Text. α and β have units of h-1 and (TCID50/ml)-1h-1, respectively. (TIFF) [file pcbi.1012264.s008.tiff]

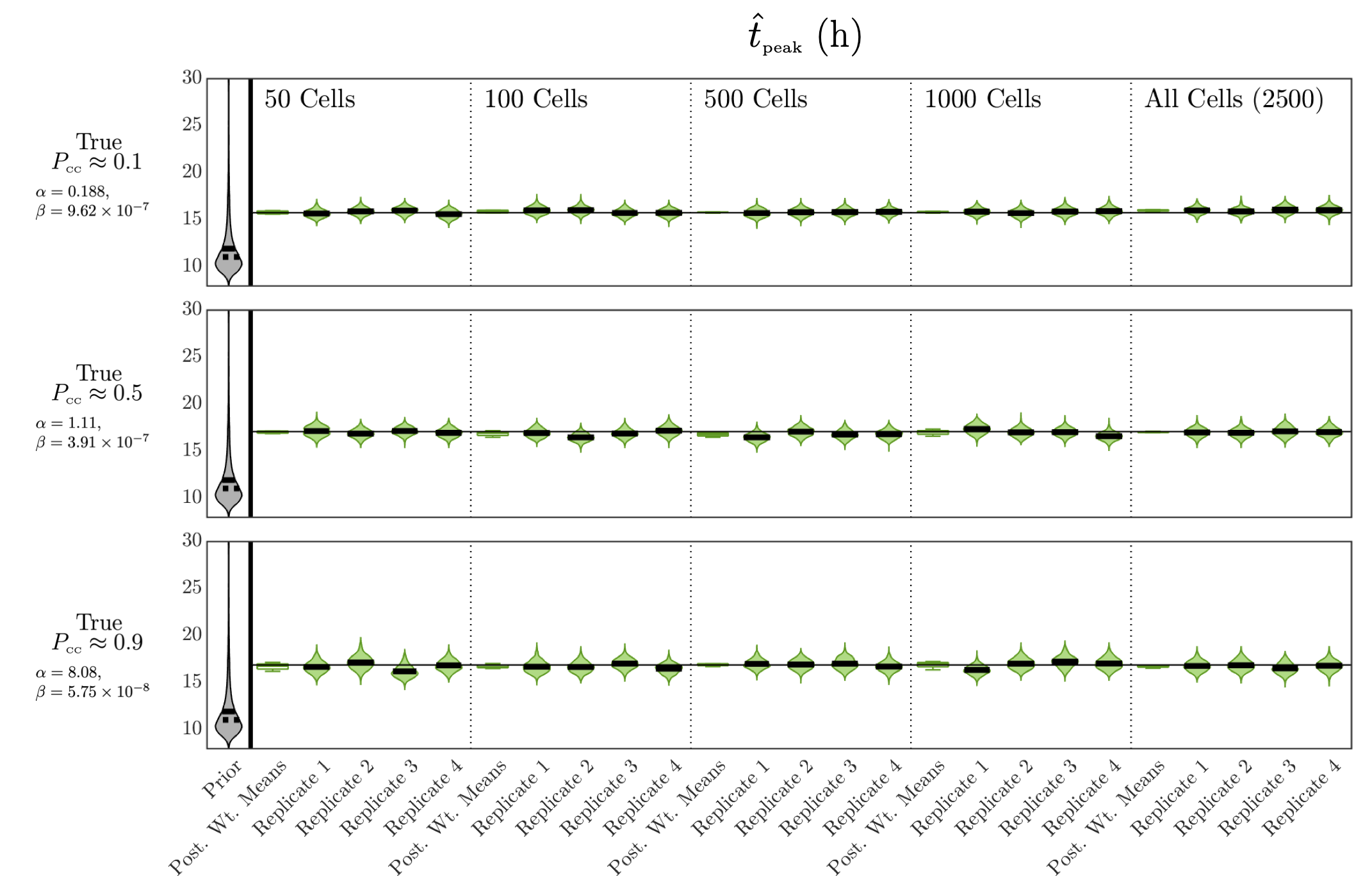

Supplement: S9 Fig — Prior density and posterior densities from individual replicates for tpeak for different values of S, the number of cells sampled to calculate the approximation κS(t) in fitting. For each value of S we also show a boxplot of the distribution of posterior weighted means across all four replicates. We show results for the case where the target values of α and β give rise to PCC values of approximately 0.1, 0.5, and 0.9 and tpeak of approximately 18h. α and β have units of h-1 and (TCID50/ml)-1h-1, respectively. (TIFF) [file pcbi.1012264.s009.tiff]

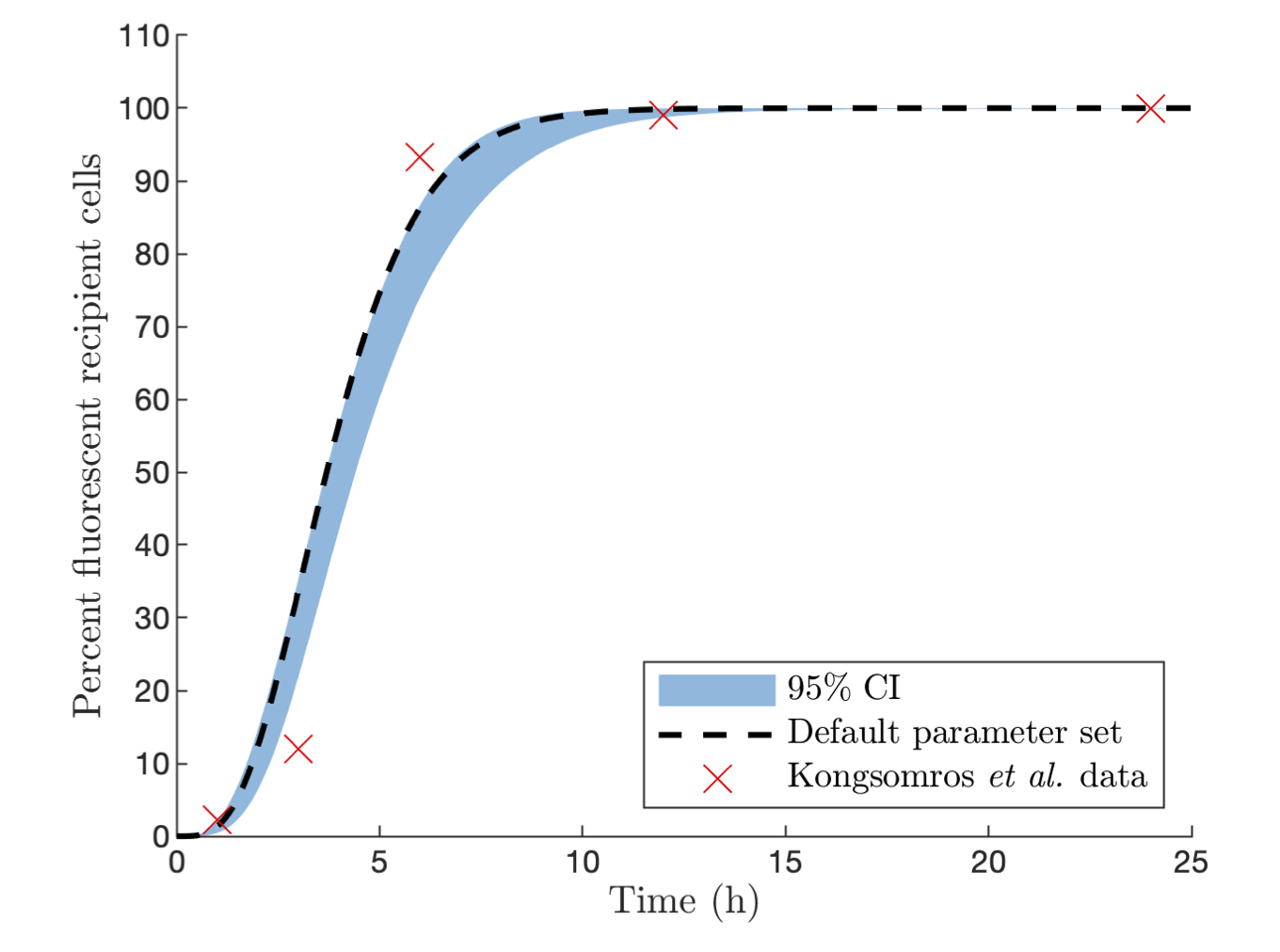

Supplement: S10 Fig — We show the 95% confidence interval of the fluorescent cell trajectories generated from the 8000 posterior samples, along with the specific trajectory of the posterior sample which we have used as our default parameter set throughout the main manuscript. (TIFF) [file pcbi.1012264.s010.tiff]
