# Supplementary material for: Spatial information allows inference of the prevalence of direct cell–to–cell viral infection
Source: PLoS Comput Biol. 2024 Jul 23;20(7):e1012264. doi: 10.1371/journal.pcbi.1012264 (PMC11296656; doi:10.1371/journal.pcbi.1012264)
Supplement: S1 Text — (PDF) [file pcbi.1012264.s011.pdf]

S1 ODE model under varying observational noise

We decided to further explore the effect of observational noise on the estimation of P_{CC} by repeating the fitting process at different values of ϕ, that is, for a variety of levels of observational noise. We do so using target parameters corresponding to a true P_{CC} of 0.5. In S1A Fig, we plot the resulting posterior distributions for P_{CC} using this process. As in the main article, we also show a box plot of the distribution of posterior medians at each level of noise, as well as the prior distribution of P_{CC} in grey. We show similar results for r estimates. S1A Fig shows that unless there is no observational noise at all, individual replicates (such as Replicate 4 in the $\phi = 10^2$ case) may result in posterior densities which are fairly compact — confident — yet centred on totally inaccurate values of P_{CC}. This can also be seen in the distribution of the replicate medians in this case, which is distributed widely with multiple outliers. Moreover, even in the case where there is no observational noise, P_{CC} posterior densities are distributed fairly widely, even though their centre is accurate to the true value of P_{CC}. This highlights the extreme sensitivity of the P_{CC} relative to fits to the model, indicating that there exist (α, β) pairs that provide a very close fit to fluorescence data, yet correspond to P_{CC} values very different to the true value.
