# Supplementary material for: Spatial information allows inference of the prevalence of direct cell–to–cell viral infection
Source: PLoS Comput Biol. 2024 Jul 23;20(7):e1012264. doi: 10.1371/journal.pcbi.1012264 (PMC11296656; doi:10.1371/journal.pcbi.1012264)
Supplement: S2 Text — (PDF) [file pcbi.1012264.s012.pdf]

S2 Spatial model under varying (artificial) observational noise

In the same manner as with the ODE model, we probed the effect of varying levels of observational noise on the data used in fitting for the spatial model. While the spatial model is inherently stochastic, as is the observational model for the spatial model, in experimental contexts there are likely additional sources of noise present in the data collection process which are not captured by our models. To explore the impact this might have on the quality of inference, we applied an additional layer of observational noise for the spatial model using the same method as for the ODE model. That is, we define the additional observational layer $f^{\text{spatial}}_{\text{artificial}}(\boldsymbol{\mathcal{D}}^{\text{spatial}}; \phi, N_{\text{sample}})$ such that if $\boldsymbol{\mathcal{D}}^{\text{spatial}} = \{\mathcal{D}^{\text{spatial}}_1, \mathcal{D}^{\text{spatial}}_2, ..., \mathcal{D}^{\text{spatial}}_m\}$, we have

$$f^{\text{spatial}}_{\text{artificial}}(\boldsymbol{\mathcal{D}}^{\text{spatial}}; \phi, N_{\text{sample}}) = \left(\frac{1}{N_{\text{sample}}}\right) \cdot \left\{\tilde{\mathcal{D}}^{\text{spatial}}_1, \tilde{\mathcal{D}}^{\text{spatial}}_2, ..., \tilde{\mathcal{D}}^{\text{spatial}}_3\right\}, \tag{S1}$$

where

$$\tilde{\mathcal{D}}^{\text{spatial}}_i \sim \textit{Negative Binomial}\left(N_{\text{sample}}\mathcal{D}^{\text{spatial}}_i, \phi\right)$$

for $i = 1, 2, ..., m$, where $N_{\text{sample}}\mathcal{D}^{\text{spatial}}_i$ and ϕ are the mean and dispersion parameter respectively of $\tilde{D}^{\text{spatial}}_i$. N_{sample} is the number of cells measured, which for simplicity we take to be 2×10^5, as we have used for the observation model for the ODE model in S1 Text. Then, given observed data $\boldsymbol{\mathcal{D}}^{\text{spatial}}_{\text{fluoro}}, \boldsymbol{\mathcal{D}}^{\text{spatial}}_{\text{cluster}}$ from the spatial model (using the usual observational model, f^{spatial}), we obtain the following noisy data

$$\left\{\tilde{\boldsymbol{\mathcal{D}}}^{\text{spatial}}_{\text{fluoro}},\ \tilde{\boldsymbol{\mathcal{D}}}^{\text{spatial}}_{\text{cluster}}\right\} = \left\{f^{\text{spatial}}_{\text{artificial}}(\boldsymbol{\mathcal{D}}^{\text{spatial}}_{\text{fluoro}}; \phi, N_{\text{sample}}),\ f^{\text{spatial}}_{\text{artificial}}(\boldsymbol{\mathcal{D}}^{\text{spatial}}_{\text{cluster}}; \phi, N_{\text{sample}})\right\}. \tag{S2}$$

Equipped with this additional observational model, we repeated the simulation–estimation process of the main article for varying levels of observational noise as in S1 Text. For a range of values for the dispersion parameter ϕ, we generated synthetic data using the composite observation model $f^{\text{spatial}}_{\text{artificial}}(f^{\text{spatial}}(\cdot))$, then otherwise carried out the parameter estimation as specified in the main article. We plot our results as posterior density distributions in S2 Fig. For each value of ϕ we indicate the posterior densities for each replicate along with a box plot of the replicate weighted mean estimates, for both P_{CC} and t_{peak}. We include the result for no artificial noise ($\phi \to \infty$) as shown in the main article as a reference. S2 Fig shows that P_{CC} and t_{peak} are consistently well–estimated for any of the examined values of ϕ; variation in the estimates away from the true value only begin to appear in the noisiest instance ($\phi = 10$). Broadly, very little loss in fit quality was acquired for any of the levels of observational noise tested. Moreover, the density of posterior replicates remains consistently compact for different noise levels, such that even for a high level of observational noise, confidence in estimated values of P_{CC} and t_{peak} remains high. Compare this to the effect of increasing observational noise for the ODE model in S1 Fig where increasing the observational noise lead to the posterior density being spread across effectively the whole range of possible values for P_{CC}.
