# Supplementary material for: Spatial information allows inference of the prevalence of direct cell–to–cell viral infection
Source: PLoS Comput Biol. 2024 Jul 23;20(7):e1012264. doi: 10.1371/journal.pcbi.1012264 (PMC11296656; doi:10.1371/journal.pcbi.1012264)
Supplement: S3 Text — (PDF) [file pcbi.1012264.s013.pdf]

S3 Assigning viral lineage at infection events in the spatial model

In the main manuscript, specifically Fig 3B, we assign to each of the initially infected cells in a simulation of the spatial model a unique identifying index j. We then, at the time a new infection takes place, assign to the newly infected cell an index corresponding to the viral lineage that infected it. For example, if we imagine an infection initiated by two infected cells with indices 1 and 2, at the time a third cell becomes infected, we determine probabilistically whether the infection arose from cell 1 or cell 2. We outline the process for determining the lineage of infections below.

Assuming that there are N_{init} lineages, we write $l(i) \in \{1, 2, ..., N_{\text{init}}\}$ for the lineage of cell i. Furthermore, we augment the ODE for the overall extracellular virus with the system

$$\frac{dV_j}{dt} = p \sum_{i=1}^{N} \frac{\mathbb{1}_{\{\sigma_i(t)=I\}} \mathbb{1}_{\{l(i)=j\}}}{N} - cV_j, \qquad \text{for } j = 1, 2, ..., N_{\text{init}}, \tag{S3}$$

where V_j is the quantity of extracellular virus in the system produced by cells of viral lineage j. Note that we have $\sum_{j=1}^{N_{\text{init}}} V_j = V$. Then, following the same argument as for assigning infection modes in the main manuscript, we define $\mathbf{E}_i^j$ as the event of an infection by viral lineage j of susceptible cell i. The probability of $\mathbf{E}_i^i$ **not** occurring (by either infection mechanism) in the time interval $[t, t + \Delta t)$ is given by

$$P(\mathbf{E}_i^j \notin [t, t + \Delta t)) = \exp\left(-\left(\alpha \sum_{j \in \nu(i)} \frac{\mathbb{1}_{\{\sigma_j(t)=I\}} \mathbb{1}_{\{l(i)=j\}}}{|\nu(i)|} + \beta V_j\right) \Delta t\right), \tag{S4}$$

where, as in the main text, $\nu(i)$ is the set of neighbours of cell i. Then we compute the probability of cell i being assigned lineage j at the time it is infected — that is, when $t = t_i^E$ — as follows

$$P(l(i) = j) = \frac{1 - P(\mathbf{E}_i^j \notin [t, t + \Delta t))}{\sum_{k=1}^{N_{\text{init}}} \left(1 - P(\mathbf{E}_i^k \notin [t, t + \Delta t))\right)}. \tag{S5}$$

As was the case when determining the mode of infection associated with a newly infected cell, we assign viral lineage as follows. First, draw a random number $x \sim \textit{Uniform}(0, 1)$, then compute

$$j^* = \min\left\{j \ : \ x < \sum_{k=1}^{j} P(l(i) = j)\right\}, \tag{S6}$$

that is, the minimum j such that the probability of cell i having an index of at most j is greater than x. Cell i is then assigned lineage j^*.
