# Supplementary material for: Spatial information allows inference of the prevalence of direct cell–to–cell viral infection
Source: PLoS Comput Biol. 2024 Jul 23;20(7):e1012264. doi: 10.1371/journal.pcbi.1012264 (PMC11296656; doi:10.1371/journal.pcbi.1012264)
Supplement: S4 Text — (PDF) [file pcbi.1012264.s014.pdf]

S4 Simulation–estimation on the spatial model using fluorescence data only

In S6A–C Figs the scatter plots of the final accepted samples in α–β space show that the posterior estimates of α and β again trace out a curve but are not necessarily concentrated near the target values. By overlaying this plot with the contours for t_{peak}, we see that the posterior distribution closely follows the contour corresponding to the (α, β) values with the same t_{peak} value as the target parameters. In S6D Fig we confirm this observation by showing violin plots of the weighted posterior densities for P_{CC} and t_{peak} across each of the replicates along with box plots of the weighted mean estimates. This figure shows that, as with the ODE model, P_{CC} is poorly estimated throughout the replicates and is prone to wide confidence intervals, while t_{peak} is very accurately recovered in each case. However, in contrast to our results from the ODE model where P_{CC} estimates in individual replicates were often compact but far from the true value, in the spatial model, we see very wide distributions of P_{CC} estimates with weighted mean values near the middle of the range of P_{CC} values. This is especially true when the P_{CC} is small. Interestingly, the posterior distributions are far more compact — and much more accurate — when the true P_{CC} is high, suggesting that P_{CC} is easier to estimate in this case, perhaps reflecting the distinct time series dynamics observed for high P_{CC}. Finally, it is also important to note that while t_{peak} represents a completely different quantity to the exponential growth rate of the ODE model, r, both parameters were very well–estimated from fluorescence time series data. This suggests that these are both good metrics for the overall rate of infection dynamics, and that this property is well–captured by the fluorescence data.
