# Supplementary material for: Spatial information allows inference of the prevalence of direct cell–to–cell viral infection
Source: PLoS Comput Biol. 2024 Jul 23;20(7):e1012264. doi: 10.1371/journal.pcbi.1012264 (PMC11296656; doi:10.1371/journal.pcbi.1012264)
Supplement: S5 Text — (PDF) [file pcbi.1012264.s015.pdf]

S5 Numerical method for the extended spatial model

For the extended spatial model, which includes a diffusive viral density, we update the discretised viral surface using an implicit–explicit finite–difference scheme. We discretise the viral density in space such that the cells themselves may be considered the nodes of the discretised surface. As a consequence, the total viral density at cell i at time τ is trivially computed as

$$\int_{S_i} V(\mathbf{x}, \tau) d\mathbf{x} = V_i^\tau,$$

where V_i^τ is the value of the discretised viral surface at node (cell) $i = 1, 2, ..., N$. In an earlier work, we discussed the discretisation of such viral surfaces, and found that when diffusion is sufficiently large compared to the length scale of the cell (greater than, say, 0.1 CD^2h^{-1}), discretisation at the cell scale was sufficient to ensure convergence of the virus PDE [1]. Throughout this work, we assume viral diffusion of at least 0.1 CD^2h^{-1}, which justifies this choice of discretisation.

For the viral diffusion, we use a Backwards–Euler method constructed on the hexagonal lattice of nodes (cells). We assume a population of N cells. The scheme for the update step is given by the matrix equation

$$\mathbf{A}\hat{\mathbf{V}}_{\text{imp}}^{\tau+\Delta t} = \hat{\mathbf{V}}^\tau, \tag{S7}$$

where

$$\hat{\mathbf{V}}^\tau = \{V_1^\tau, V_2^\tau, ..., V_N^\tau\}, \tag{S8}$$

and $\mathbf{A}$ is the $N \times N$ discretised diffusion matrix, which reflects the adjacency structure of the nodes, such that

$$\mathbf{A}_{i,j} = \begin{cases} \left(1 + \dfrac{4D\Delta t}{\Delta x^2}\right), & i = j, \ j = 1, 2, ..., N, \\ -\dfrac{2}{3}\dfrac{D\Delta t}{\Delta x^2}, & i \in \nu(j), \ j = 1, 2, ..., N, \\ 0, & \text{otherwise.} \end{cases}$$

Here, Δx is the distance between cell centres (cell diameter, or CD), and $\nu(i)$ is the set of cells (nodes) neighbouring cell (node) i, as defined in the main manuscript. Recall we apply toroidal periodic boundary conditions, such that $|\nu(i)| = 6$ for $i = 1, 2, ..., N$. Throughout the manuscript, we work in units of CD, and hence take $\Delta x = 1$. In an update step, we compute the value of the discretised virus surface at time $\tau + \Delta t$ from Equation (S7) using sparse system solvers.

Having computed the viral diffusion step, we then apply an explicit scheme for the remaining terms of the virus PDE:

$$\mathbf{V}_{\text{exp}}^{\tau+\Delta t} = \mathbf{V}^\tau + \Delta t\left(\frac{2p}{\sqrt{3}\Delta x^2}\mathbf{I}^\tau - c\mathbf{V}^\tau\right), \tag{S9}$$

where

$$\mathbf{I}^\tau = \left\{\mathbb{1}_{\{\sigma_1(\tau)=I\}}, \mathbb{1}_{\{\sigma_2(\tau)=I\}}, ..., \mathbb{1}_{\{\sigma_N(\tau)=I\}}\right\}.$$

The final update step, then, is given by

$$\mathbf{V}^{\tau+\Delta t} = \mathbf{V}_{\text{imp}}^{\tau+\Delta t} + \mathbf{V}_{\text{exp}}^{\tau+\Delta t}. \tag{S10}$$

As described in the main text, values for α and β for varying viral diffusion coefficients were calculated from lookup tables. We list the computed values for a range of values for D in Table A.

	$P_{\text{CC}} \approx 0.1$		$P_{\text{CC}} \approx 0.5$		$P_{\text{CC}} \approx 0.9$	
D	α	β	α	β	α	β
10^{-1}	0.845	3.476×10^{-5}	5.90	2.49×10^{-5}	17.7	9.08×10^{-6}
$10^{-0.5}$	0.575	5.79×10^{-6}	4.0827	3.70×10^{-6}	17.0	8.03×10^{-7}
10^{0}	0.321	1.75×10^{-6}	2.51	1.06×10^{-6}	13.44	2.07×10^{-7}
$10^{0.5}$	0.197	9.51×10^{-7}	1.54	5.37×10^{-7}	10.0	9.50×10^{-8}
10^{1}	0.165	7.55×10^{-7}	1.16	4.00×10^{-7}	7.81	6.97×10^{-8}
$10^{1.5}$	0.148	6.94×10^{-7}	1.04	3.62×10^{-7}	6.90	6.02×10^{-8}
10^{2}	0.145	6.97×10^{-7}	1.00	3.56×10^{-7}	6.18	6.27×10^{-8}
∞	0.188	9.62×10^{-7}	1.11	3.91×10^{-7}	8.08	5.75×10^{-8}

Table A: α and β values for varying extracellular viral diffusion and P_{CC}. α and β values for the specified values of the extracellular viral diffusion coefficient D and P_{CC} as deduced from our lookup tables. In each case the infected peak time is fixed at 18h. α has units h^{-1}, β has units $(\text{TCID}_{50}/\text{ml})^{-1}\text{h}^{-1}$, D has units CD^2h^{-1}.

References

[1] Williams T, McCaw JM, Osborne JM. Choice of spatial discretisation influences the progression of viral infection within multicellular tissues. Journal of Theoretical Biology. 2023;573:111592. doi:https://doi.org/10.1016/j.jtbi.2023.111592.
