# Supplementary material for: Spatial information allows inference of the prevalence of direct cell–to–cell viral infection
Source: PLoS Comput Biol. 2024 Jul 23;20(7):e1012264. doi: 10.1371/journal.pcbi.1012264 (PMC11296656; doi:10.1371/journal.pcbi.1012264)
Supplement: S6 Text — (PDF) [file pcbi.1012264.s016.pdf]

S6 Parameter estimation for the ODE model

In order to obtain a set of realistic parameters for the spread of influenza, we carried out a Bayesian parameter estimation for the ODE model (as defined in the main manuscript) using experimental data from Kongsomros *et al.* [1]. We fit all of the parameters of the model simultaneously using a No U–Turn Sampling (NUTS) Markov Chain Monte Carlo (MCMC) algorithm [2], with the exception of the number of latent compartments, K, since discrete parameters were not well–handled by the algorithm. We determined that $K = 3$ provided a reasonable fit to the data. For the remaining parameters, we used the following prior distributions:

$$\begin{aligned}
\alpha &\sim \textit{Truncated Normal}(0, \alpha_{\text{ref}}),\\
\beta &\sim \textit{Lognormal}(\log(\beta_{\text{init}}), 1),\\
\gamma &\sim \textit{Truncated Normal}(0, \gamma_{\text{ref}}),\\
\delta &\sim \textit{Lognormal}(\log(\delta_{\text{init}}), 1),\\
\log(p) &\sim \textit{Truncated Normal}(0, \log(p_{\text{ref}})),\\
c &\sim \textit{Lognormal}(\log(c_{\text{init}}), 1),
\end{aligned}$$

with $\alpha_{\text{ref}} = 4\ \text{h}^{-1}$, $\beta_{\text{init}} = 2.167 \times 10^{-6}(\text{TCID}_{50}/\text{ml})^{-1}\text{h}^{-1}$, $\gamma_{\text{ref}} = 10\text{h}^{-1}$, $\delta_{\text{init}} = 0.217\text{h}^{-1}$, $\log(p_{\text{ref}}) = 10$, $c_{\text{init}} = 0.3125\text{h}^{-1}$. For β, δ and c, which are difficult to estimate from the available data, we used lognormal priors centred on parameter estimates adapted from Baccam *et al.* [3], with the additional assumptions that, given the inclusion of an additional mode of infection, the rate of extracellular viral decay c would be elevated, and the cell–free infectivity β would be reduced substantially (we reduced the assumed β by three orders of magnitude). For the remaining parameters, we used wide normal distributions centred at zero and truncated to be positive. We used the likelihood

$$N_{\text{sample}^*}\mathcal{D}_i^{\text{exp}} \sim \textit{Binomial}(N_{\text{sample}^*}, F(t_i)), \tag{S11}$$

where, following similar notation to the main manuscript, $\mathcal{D}_i^{\text{exp}}$ is the observed proportion of fluorescent cells in the experimental data at time point t_i and $F(t_i)$ is the predicted proportion of fluorescent cells at the same time using the model. Since, in the published data, the fluorescent cell proportion was computed by manual counting [1], we assumed N_{sample^*} was the size of the subset of the overall cell population (approximately 200,000, as quoted in the main manuscript) sampled for manual counting. Note that this formulation is somewhat different to how we have defined the data collection process in the main manuscript, which assumed analysis of the entire cell population with overdispersed noise. The latter formulation better reflects systematic sample processing, such as by flow cytometry, which is a more typical approach in the biological literature. Since the sample size used in Kongsomros *et al.* for manual counting was not available, we assumed $N_{\text{sample}^*} = 100$, which resulted in a reasonable degree of observational noise without imposing artificial additional noise.

We ran two chains using the inference algorithm which were observed to mix well. For both chains we drew 5000 samples and discarded the first 1000 as burn–in. We randomly selected one of the accepted samples as a default parameter set which we use in the main manuscript and also list below in Table A. Table A shows these default parameters offer reasonable agreement with the estimates from Baccam *et al.* [3]. In S10 Fig we generate fluorescent cell time series curves for each of the 8000 accepted parameter samples and plot their 95% confidence interval against the data. We also show the specific trajectory for the default parameter sample we used throughout the manuscript. S10 Fig shows that the model provides good agreement with the experimental data, including with the default parameter set.

Description	Symbol	Default fitted value	Baccam *et al.* estimate	Units
Cell–to–cell infectivity	α	9.502707×10^{-1}	*	h^{-1}
Cell–free infectivity	β	1.3×10^{-6}	2.167×10^{-3}	$(TCID_{50}/ml)^{-1}h^{-1}$
Number of delay compartments	K	3	+	
Eclipse cell activation rate	γ	3.366934×10^{-1}	1.67×10^{-1}	h^{-1}
Death rate of infected cells	δ	8.256588×10^{-2}	2.17×10^{-1}	h^{-1}
Extracellular virion production rate	p	1.321886×10^{6}	7.68×10^{5}	$(TCID_{50}/ml)\ h^{-1}$
Extracellular virion clearance rate	c	4.313531×10^{-1}	2.17×10^{-1}	h^{-1}

Table A: Default fitted model parameters compared to literature estimates. The default parameter set obtained from our parameter estimation, compared to previous estimates published by Baccam *et al.* [3]. * Baccam *et al.* only had the cell–free mode of infection. + Baccam *et al.* considered only a single delay compartment.

References

[1] Kongsomros S, Manopwisedjaroen S, Chaopreecha J, Wang SF, Borwornpinyo S, Thitithanyanont A. Rapid and Efficient Cell-to-Cell Transmission of Avian Influenza H5N1 Virus in MDCK Cells Is Achieved by Trogocytosis. Pathogens. 2021;10(4). doi:10.3390/pathogens10040483.

[2] Stan Development Team. Stan Modeling Language Users Guide and Reference Manual, Version 2.33 (R). https://mc-stan.org.; 2023.

[3] Baccam P, Beauchemin C, Macken CA, Hayden FG, Perelson AS. Kinetics of Influenza A Virus Infection in Humans. Journal of Virology. 2006;80(15):7590–7599. doi:10.1128/JVI.01623-05.
