# Supplementary material for: Spatial information allows inference of the prevalence of direct cell–to–cell viral infection
Source: PLoS Comput Biol. 2024 Jul 23;20(7):e1012264. doi: 10.1371/journal.pcbi.1012264 (PMC11296656; doi:10.1371/journal.pcbi.1012264)
Supplement: S7 Text — (PDF) [file pcbi.1012264.s017.pdf]

S7 PMC algorithm for parameter estimation using the spatial model – fluorescence data only.

PMC algorithm for parameter estimation using the spatial model – fluorescence data only.

Input: Model $\mathcal{M}(\alpha, \beta)$, prior distributions for target parameters $\pi_\alpha(\alpha)$ and $\pi_\beta(\beta)$, target number of particles N_P, number of generations G, reference data $\boldsymbol{\mathcal{D}}^{\text{spatial}}$, distance metric $d(\cdot,\cdot)$, perturbation kernel $K(\cdot|\cdot)$, initial acceptance proportion $p_{0,\text{accept}}$, threshold tightening parameter q.
Output: Weighted samples from the posterior distributions $\hat{\pi}_\alpha(\alpha|\boldsymbol{\mathcal{D}}^{\text{spatial}})$, $\hat{\pi}_\beta(\beta|\boldsymbol{\mathcal{D}}^{\text{spatial}})$.

Rejection sampling
for $i = 1, 2, ..., \lceil N_P/p_{0,\text{accept}} \rceil$ **do**
  Randomly draw $\hat{\alpha}_i$ and $\hat{\beta}_i$ from $\pi_\alpha(\alpha)$ and $\pi_\beta(\beta)$, respectively.
  Obtain the model output using these parameters, $\hat{\boldsymbol{\mathcal{D}}}^{\text{spatial},(i)} = \mathcal{M}(\hat{\alpha}_i, \hat{\beta}_i)$.
  Compute the distance between model output and reference data $\epsilon_i = d(\hat{\boldsymbol{\mathcal{D}}}^{\text{spatial},(i)}, \boldsymbol{\mathcal{D}}^{\text{spatial}})$.
end for
Set $\mathcal{I}_1, \mathcal{I}_2, ..., \mathcal{I}_{N_P}$, as the set of indices i corresponding to the smallest N_P values of the ϵ_is.
for $j = 1, 2, ..., N_P$ **do**
  Set $\mathcal{P}_j = (\hat{\alpha}_{\mathcal{I}_j}, \hat{\beta}_{\mathcal{I}_j})$.
  Set $w_j = 1/N_P$.
end for
$\mathcal{P} = \{\mathcal{P}_1, \mathcal{P}_2, ..., \mathcal{P}_{N_P}\}$ is the initial **particle** population. $w = \{w_1, w_2, ..., w_{N_P}\}$ is the initial **weight** vector. Set the distance threshold ϵ_D as the qth quantile of the ϵ_is.

Importance sampling
for $g = 1, 2, ..., G$ **do**
  Set number of accepted particles $N_{\text{accepted}} \leftarrow 0$
  while $N_{\text{accepted}} < N_P$ **do**
    Randomly draw a particle $\mathcal{P}_j$ with probability w_j.
    Perturb particle by the kernel $K(\cdot|\mathcal{P}_j)$ to obtain a new sample $(\hat{\alpha}, \hat{\beta})$.
    Obtain the model output using these parameters, $\hat{\boldsymbol{\mathcal{D}}}^{\text{spatial},(i)} = \mathcal{M}(\hat{\alpha}_i, \hat{\beta}_i)$.
    Compute the distance between model output and reference data $\epsilon_i = d(\hat{\boldsymbol{\mathcal{D}}}^{\text{spatial},(i)}, \boldsymbol{\mathcal{D}}^{\text{spatial}})$.
    if $\epsilon_i < \epsilon_D$ **then**
      Set $N_{\text{accepted}} \leftarrow N_{\text{accepted}} + 1$ and $\mathcal{P}^{\text{next}}_{N_{\text{accepted}}} = (\hat{\alpha}_i, \hat{\beta}_i)$.
    else
      Return to start of **while**.
    end if
  end while
  for $i = 1, 2, ..., N_P$ **do**
    Set $w_i^{*,\text{next}} = w_i / \sum_{j=1}^{N_P} K\left(\mathcal{P}_i^{\text{next}}|\mathcal{P}_j\right) w_j$
  end for
  Set $\mathcal{P} \leftarrow \left\{\mathcal{P}_1^{\text{next}}, \mathcal{P}_2^{\text{next}}, ..., \mathcal{P}_{N_P}^{\text{next}}\right\}$, $w \leftarrow (1/\sum_{i=1}^{N_P} w_i^{*,\text{next}}) \cdot \left\{w_1^{*,\text{next}}, w_2^{*,\text{next}}, ..., w_{N_P}^{*,\text{next}}\right\}$
  Set the distance threshold ϵ_D as the qth quantile of the ϵ_is.
end for
