# Supplementary material for: Spatial information allows inference of the prevalence of direct cell–to–cell viral infection
Source: PLoS Comput Biol. 2024 Jul 23;20(7):e1012264. doi: 10.1371/journal.pcbi.1012264 (PMC11296656; doi:10.1371/journal.pcbi.1012264)
Supplement: Attachment — Submitted filename: ResponseToReviewers.pdf [file pcbi.1012264.s018.pdf]

Response to reviewers comments

We would like to thank the editors and reviewers for their very helpful comments, suggestions and insights. They have certainly helped improve the paper.

Below we have systematically addressed each of the reviewers comments. We hope that our replies along with the modifications to the paper — some of which represent substantial improvements — are sufficient to address the concerns of the reviewers.

In this response we have presented the reviewers comments using quotes presented in *italicised* text and our responses are given in plain text. Details of changes to the manuscript in response are given as a bullet list in this document and these changes are tracked in the manuscript. In both instances, added text is shown in red. This document cites the same bibliography as the revised manuscript.

Editor comments

> *As you will see, two of the reviewers expressed substantial concerns about the study, and indicated that the approach might not be worked out sufficiently well and that therefore the conclusions are not sufficiently supported by the analysis. One thing I noticed before sending out the paper for review was that some of the literature that aimed to measure the relative contribution of cell free vs cell-to-cell transmission in HIV infection has not been cited, which should be corrected in future versions. This includes:*
>
> *- Komarova NL, Anghelina D, Voznesensky I, Trinité B, Levy DN, Wodarz D. Relative contribution of free-virus and synaptic transmission to the spread of HIV-1 through target cell populations. Biology letters. 2013 Feb 23;9(1):20121049.*
>
> *- Kreger J, Garcia J, Zhang H, Komarova NL, Wodarz D, Levy DN. Quantifying the dynamics of viral recombination during free virus and cell-to-cell transmission in HIV-1 infection. Virus evolution. 2021 Jan;7(1):veab026.*

We thank the editor for his comments, which brought to light some publications which we were not aware of. Our literature search primarily concentrated on viral infections with a notable spatial structure, such as influenza and hepatitis viruses, and less so on HIV, which, since the target cells are motile, has more well–mixed dynamics which are well–described by ODE models. This makes the methodology which we develop here less relevant in the case of HIV (as Reviewer 3 pointed out). Nonetheless, the original draft of our manuscript neglected some important techniques and results from the HIV literature which add important context to our work. We have revised the introduction and discussion sections of our manuscript to include discussion of these works and explain why our work is somewhat distinct from some HIV studies. We list these changes below:

- Line 15 (revised manuscript): Adjusted text in Introduction, additions in red.

  Over the last decade, a substantial quantity of experimental and modelling studies have attempted to quantify the relative contributions of the cell–to–cell and cell–free mechanism in infection with different viral species. Among these works, the most developed body of literature concerns HIV. Interdisciplinary studies led by Komarova [20] and Iwami [18] suggested that cell–to–cell and cell–free infections contribute roughly equally in HIV infection *in vitro*; more recent work by Kreger and colleagues [22], which also modelled the latent stage of infection in HIV, inferred a significantly higher rate of cell–free infection. In hepatitis C, modelling efforts led by Graw and Durso–Cain suggested that cell–free infection events were rare, yet worked synergistically with the cell–to–cell infection strategy to rapidly accelerate the overall rate of infection spread [13, 8]. Blahut and coworkers used modelling to quantify the proportion of the two modes of spread using *in vitro* experimental data, and claimed that as little as 1% of the infection events observed were due to cell–to–cell infection [3]. Experimental work by Kongsomros and colleagues suggested that the proportion of cell–to–cell infections in influenza was very low, but elevated in more pathogenic strains of the virus

[21]. Experimental work in SARS-CoV-2 by Zeng and collaborators claimed that cell–to–cell infection represented around 90% of infections [39].

These estimates in the literature for the relative contribution of cell–to–cell spread in infection are subject to substantial uncertainty, and share the common limitation that they rely on experiments which block one of the modes of viral spread, compared to a control case where both routes of infection are active [20, 18, 22, 3, 21, 39]. This inhibition can be implemented in a number of ways, such as by conducting infection assays in the presence of an antiviral agent or a physical barrier to viral diffusion like methylcellulose to block cell–free infection [21, 39], or by constantly shaking the cell culture to prevent the formation of virological synapses which enable cell–to–cell infection, in the case of HIV [20, 18, 22]. These approaches, however, share some common limitations. For one, the two modes of viral spread are known to interact synergistically, and the inhibition of one of the infection mechanisms invariably influences the strength of the other mode of infection [20, 18, 15]. For instance, in the case of the static and shaking assays for HIV infection described above, Komarova and colleagues estimated that shaking the cell culture increased the rate of cell–free infection by around 1.33 times [20]. A second shortcoming of this approach is its inapplicability to *in vivo* settings. In living organisms, host toxicity or simple practicality prevents the use of most interventions to block one mode of viral spread, such as treating cells with methylcellulose or continuously shaking the cell population, yet the relative contribution of the two modes of infection may be substantially different *in vivo* compared to in cell culture. For instance, Dixit and Perelson estimated that in human hosts, roughly 90% of infection was due to cell–to–cell spread [7], whereas estimates from *in vitro* data placed this figure at around 50% [20, 18, 22].

Only a few studies have attempted to infer the balance of the two modes of infection spread from data where both mechanisms are unimpeded. Imle and collaborators studied HIV infection in cell cultures embedded either in suspension or a 3D collagen scaffold, and calibrated an ODE model to the data to attempt to infer the relative contribution of the two modes of infection from virion and cell count data [17]. The authors suggested that the inference implied almost all infection in the suspension was due to cell–free infection, however, the 95% confidence interval for the proportion of cell–free infection encompassed virtually the whole range from 0 to 100% [17]. In hepatitis C, Kumberger and colleagues demonstrated modifications that can be made to a standard ordinary differential equation (ODE) model of viral dynamics in order to better describe cell-to-cell infection, but were nonetheless unable to satisfactorily infer the prevalence of cell-to-cell infection from synthetic data where the two modes of infection occurred simultaneously [24]. The authors moreover did not examine whether estimates of this quantity were improved or weakened when the true balance of the two mechanisms in the synthetic data was changed [24]. The limits of identifiability of the proportion of cell-to-cell infection — under different conditions, using different models, and based on different sources of observational data — has not been systematically studied.

Here, we conduct simulation–estimation studies using two mathematical models for viral infections with two modes of spread: one non-spatial ODE system and one spatially-explicit multicellular model. In both cases, we generate synthetic data using the model in combination with an observational model, and attempt to re-estimate the prevalence of cell-to-cell infection from the resulting observations. We repeat this process under a range of conditions and with different types of available data for fitting. Our results provide an important background for the practical identifiability of the cell-to-cell infection prevalence, and offer guidance for the design of models and experimental systems best equipped to learn this quantity. It is important to mention that the analysis which we conduct here is limited to infections of static tissues, and does not extent to infections in motile cell populations, such as HIV. Since, in this case, the migration of target cells enables well–mixed conditions, there is no notable spatial structure to infection and thus the resulting dynamics are less easily distinguished.

- Line 522 (revised manuscript): Adjusted text in Discussion, additions in red.

  Our work is not the first in the literature to attempt to quantify the relative roles of cell–free and cell–to–cell infection routes. A number of mathematical modelling publications [24, 8, 3, 13, 17, 20, 18], along with experimental works [39, 21] have applied varying models and methods to determine the prevalence of cell–to–cell infection. A common theme among the majority of these works is the use of data collected from infections where one mode of infection is inhibited: either the cell–to–cell mechanism [20, 22, 18], or the cell–free mechanism [39, 21, 3]. This approach has substantial limitations. For one, this inhibition process may either restrict or enhance the efficacy of the other mode of infection, either directly or by interrupting the synergistic relationship between the two mechanisms, as we discussed in the Introduction [20, 18, 15]. This approach is moreover limited to *in vitro settings*.

  The alternative approach — collecting data from experiments in which both modes of infection are unimpeded — raises additional challenges, but is more robust and, since it requires less invasive experimental intervention, dramatically widens the scope of experiments able to be used for inference. However, earlier estimates of the proportion of infections from the two modes of spread using this data have been subject to substantial uncertainty [13, 17, 24]. Kumberger and collaborators used a spatial model with two modes of infection to generate synthetic global observational data (similar to the fluorescence data we have used here) and attempted to fit it using ODE models [24]. As we have found here, their work suggested that models which (artificially) account for the spatial structure of infection provided better estimates of the prevalence of cell–to–cell spread P_{CC}. However, even then, these estimates were still not especially accurate and were subject to systematic biases, even when fitting multiple observational datasets in a single fit. Another study by Imle and colleagues also calibrated an ODE model with two modes of spread to experimental viral load and infected cell count data from an *in vitro* HIV system, and encountered confidence intervals for the proportion of cell–to–cell infection ranging almost all the way from 0–100% [17]. Our work provides context for these findings, offers novel insight on the practical identifiability of P_{CC}, and suggests an improved method for determining this quantity. We showed that ODE systems were unable to identify P_{CC}, even when fitting data generated by the system itself, and moreover showed that the collection of spatial information, in the form of the clustering metric $\kappa(t)$, was necessary to learn P_{CC}, even with a spatial model.

- Line 491 (revised manuscript): Added text in Discussion, additions in red:

  Another important simplification in our approach was our implementation of a global extracellular virus population in the spatial model, rather than a spatially-explicit, diffusing viral population. It is important to clarify here that our use of a global extracellular viral population is based on an assumption of rapid viral transport. This is an important distinction from the modelling literature on HIV (e.g. [17, 14, 20, 18]), where the system can be characterised by well–mixed dynamics since the target cells are also motile. This fact substantially changes the mode of action of the cell–to–cell mechanism and thus also the spatial structure of the infection. For this reason, the methods we have developed here do not extend to HIV infection.

Reviewer 1 comments

The authors present a pair of mathematical models of infection which allow for both free virus and cell-cell transmission, model the measurement noise assuming cellular fluorescence as a determinant of infection, and then numerically simulate the estimation of the cell-cell and cell-virus transmission rates assuming that all other rates are known. The authors show that if spatial information is not gathered (the experiment is assumed to be well-mixed), the resulting parameter estimates have very broad posterior

distributions, and include regions where the estimate appears quite confident even when it is incorrect. However, when spatial data is collected (assuming transmission on a 2D culture from a known initial infection point), the posterior distribution of the parameter estimates is constrained and the confidence interval consistently contains the true value.

This is a well-written paper addressing an important topic not just in viral dynamics but also in cell signaling. My only concern is that the authors are only addressing practical identifiability, and they do so without specifically stating this. I would ask them to explicitly state that their conclusions are that alpha and beta are not practically identifiable in the non-spatial system with realistic levels of noise. I would also be interested to know whether the parameters in question are structurally identifiable in equations 1-11, though I wouldn't insist on this for this paper.

We thank the reviewer for their comment and for pointing out an important nuance that was missing from our discussion. We feel that, since the focus of our paper is primarily numerical and simulation–based in nature, a rigorous structural identifiability analysis would be beyond the scope of our work we agree that such an analysis would be very useful in revealing the limits of identifiability for not just α and β in these models, but potentially the other parameters as well (which also influence P_{CC}). Nonetheless, we have modified our text to indicate that our results are only a practical identifiability analysis and indicated the need for a structural identifiability analysis also. We outline our changes to the manuscript below:

- Line 145 (revised manuscript): Adjusted text in Results, additions in red.

  Figure 2d shows that while r is well estimated compared to its prior distribution — regardless of the choice of target parameters — P_{CC} cannot be practically identified, even when a conservative amount of observational noise is present.

- Line 467 (revised manuscript): Adjusted text in Results, additions in red.

  There are additional identifiability concerns attached with estimating the other parameters — the cell-free infection rate β and extracellular viral production rate p, for instance, are well known to only be determined as a product [29, 16] — and it is possible that estimating these additional parameters may introduce further complications in determining P_{CC}. Moreover, our work has presented a *practical* identifiability analysis of our model systems, and not a *structural* identifiability analysis. For the sake of simplicity, as well as constraints on computational complexity, we have not carried out a structural identifiability analysis in this work, however this investigation in future will provide further insights into the use of mathematical models in the inference of the prevalence of cell–to–cell infection.

Reviewer 2 comments

In this article, the author address the question what type of information might be necessary to reliably infer cell-to-cell transmission dynamics for viral infections. Using a theoretical approach by simulating viral spread with population-based ODE and spatially resolved agent-based models, they show that additional spatial information on mean clustering dynamics of infection can improve the inference of cell-to-cell transmission dynamics in the context of the simultaneous appearance of cell-free and cell-to-cell viral transmission modes.

The study targets a well-defined problem with a clear focus. It is well written and thoroughly structured, although some technical aspects could need some further clarification. The study points towards a useful spatial quantity that could be determined in order to improve inference of cell-to-cell transmission dynamics. However, it is not

clear how suitable this approach would be in general, and how applicable it might be given that some aspects influencing these dynamics have not been investigated.

Comment 2.1

An important aspect which does not seem to be investigated concerns the diffusion rate of the virus. Dependent on fast or slow diffusion, the proposed clustering metric could potentially be impaired in the ability to dissect cell-free and cell-to-cell transmission dynamics. In order to evaluate the general applicability of this single spatial metric in context of viral infections, it might be recommended to investigate how sensitive the inference of the transmission modes would be with regard to the viral diffusion parameter.

The reviewer makes an excellent point which is probably the most substantial limitation of our original manuscript. Our choice to approximate the diffusion of virions as infinite, which is in line with several other models of the two modes of viral spread [3, 12, 17], was originally made for several reasons. For one, we used a fairly small patch of model cells which should reduce the sensitivity to the rate of viral diffusion, and moreover, we assumed from a phenomenological point of view that "nearby" infections were mainly caused by the cell–to–cell route, whereas long–range infection was due to cell–free infection. From a practical perspective, avoiding diffusion in our model system also substantially reduces computational cost. Nonetheless, we agree with the reviewer that this is a strong and not entirely biological assumption, and one which might be more or less applicable to different experimental systems where the rate of free virus diffusion might vary. As such, we have introduced a substantial new section to our study in order to explore the sensitivity of our results to the value of the viral diffusion parameter. Broadly, we have done so by generating synthetic observation data using an extended version of the spatial model which incorporates finite viral diffusion of varying rates, and re-estimating the infectivity parameters using the (misspecified) instantaneous diffusion model. We find that the misspecified model is sufficient to obtain reasonable estimates of the prevalence of cell–to–cell infection provided the diffusion is reasonably fast (diffusion coefficients consistent with Stokes–Einstein estimates for influenza or SARS–CoV–2 virions in water or plasma at relevant temperatures are sufficient). At lower rates of diffusion, the quality of inference is reduced, but the inference may still be useful as an upper bound on the prevalence of cell–to–cell infection. We outline our changes to the manuscript to accommodate this addition below. Note that, to avoid confusion between the extracellular viral diffusion coefficient, D, and for observational data points, we have adjusted the notation for the latter from $\mathbf{D} = \{D_1, D_2, ..., D_m\}$ to $\boldsymbol{\mathcal{D}} = \{\mathcal{D}_1, \mathcal{D}_2, ..., \mathcal{D}_m\}$ throughout the manuscript and the Supplementary Information.

- **New result Section "The proportion of cell–to–cell spread can be inferred from diffusion–limited observational data within reasonable limits"**
  Refer to revised manuscript
- **Revised Methods Sub-section "A multicellullar spatial model for dual–spread dynamics"**
  Refer to revised manuscript
- **New Supplementary Section "Numerical method for the extended spatial model"**
  Refer to revised Supplementary Information
- **New Supplementary Section "$\kappa(t)$ for varying diffusion coefficients at fixed values of P_{CC}"**
  Refer to revised Supplementary Information
- **New Supplementary Section "Effect of extracellular viral diffusion parameter in observational data on estimates of $\hat{t}_{\mathrm{peak}}$"**
  Refer to revised Supplementary Information

- Line 442 (revised manuscript): Added text in Discussion.

  Since our spatial model assumed uniform extracellular virus (corresponding to infinitely fast viral diffusion) we tested whether the approach outlined here would apply when observational data was obtained with diffusion–limited viral spread. We generated synthetic fluorescence and clustering observational data using an extended spatial model with finite viral diffusion of varying rates and re–estimated P_{CC} and t_{peak} using the misspecified uniform extracellular virus model. Despite not accounting for finite diffusion, the simulation–estimation provided reasonable estimates of both P_{CC} and t_{peak} provided the diffusion coefficient was at least around 10 $\mathrm{CD}^2\mathrm{h}^{-1}$, which is lower than the Stokes–Einstein estimate for diffusion of influenza or SARS–CoV–2 virions in body plasma at body temperature [1, 16]. At lower values of the diffusion coefficient ($D = \mathcal{O}(1)$ $\mathrm{CD}^2\mathrm{h}^{-1}$), estimates lose accuracy and incur a substantial bias, but the simulation–estimation may still offer qualitative upper bounds on the prevalence of cell–to–cell infection. It is therefore of great interest what the true value of the extracellular viral diffusion coefficient is under given conditions. Unfortunately, this quantity is not known. Sego and colleagues, for instance, provided a plausible range for the diffusion coefficient of SARS–CoV–2 virions in lung mucus which spanned six orders of magnitude [32]. Reflecting this uncertainty, in our analysis here we have explored a wide range of biologically reasonable diffusion coefficients and obtained useful inferences for a realistic interval of values.

- Line 491 (revised manuscript): Added text in Discussion, additions in red.

  Another important simplification in our approach was our implementation of a global extracellular virus population in the spatial model, rather than a spatially-explicit, diffusing viral population. It is important to clarify here that our use of a global extracellular viral population is based on an assumption of rapid viral transport. This is an important distinction from the modelling literature on HIV (e.g. [17, 14, 20, 18]), where the system can be characterised by well–mixed dynamics since the target cells are also motile. This fact substantially changes the mode of action of the cell–to–cell mechanism and thus also the spatial structure of the infection. For this reason, the methods we have developed here do not extend to HIV infections. We observed that for values of the viral diffusion coefficient far smaller than the Stokes–Einstein estimate ($\mathcal{O}(0.1)$ $\mathrm{CD}^2\mathrm{h}^{-1}$), our inference fails. It is highly likely that the increased viscosity of lung mucus and other obstacles *in vivo* are likely to restrict the spread of free virions within the host compared to the Stokes–Einstein estimate [17], and, although the extent of this is unknown, diffusion coefficients in this range have been used by other authors [32, 1]. As such, based on the best quantitative information available, the inference approach outlined here is likely to provide useful estimates of the proportion of cell–to–cell infection, however, should the actual diffusion coefficient turn out to be significantly smaller, this would substantially increase the difficulty of the inference problem. Fitting with a model which accounts for finite viral diffusion could offer an improved fit to the data. We saw in Figure 5g that there is still substantial variation in clustering metrics for changes in P_{CC}, even at very low diffusion coefficients. However, such a model would come at a substantially increased computational cost, and would require the diffusion coefficient D to be estimated along with α and β (if not also the other parameters of the model), significantly adding to the number of simulation iterations needed to fit the model. It is moreover not clear *a priori* how well the diffusion coefficient would be estimated or how estimates of P_{CC} would be influenced by inaccurate estimation of the diffusion coefficient.

- Line 574 (revised manuscript): Added text in Discussion, additions in red.

  We found that when an additional data source, describing the clustering structure of the infection, was also used for fitting, P_{CC} could be accurately determined using the spatial model. This was the case even when the clustering metric was only approximated using a small sample of the cell sheet, or when the model was fit to observational data with realistic constraints on the diffusion of free virions.

Comment 2.2

> *The study focuses on the inference of cell-to-cell transmission dynamics within solid epithelial tissue in the context of a target cell limited model. This analysis would neglect cell proliferation and tissue regeneration dynamics, which might influence local cellular densities and, thus, cell interaction dynamics and the growth of infected foci by cell-to-cell transmission. The authors carefully acknowledge the limitations of their approach within the discussion, but the ability to practically infer viral transmission dynamics from undisturbed cultures with a single spatial metric might be impaired given the additional sources of variation. It might be interesting to determine in this simplified model of viral infection dynamics how much observational noise would be actually tolerable and still allowing inference. This could point towards the general applicability of the method with regard to the various aspects (cell heterogeneity, proliferation dynamics, etc) that could impair clear spatial patterns mediated by a given transmission profile.*

The reviewer makes a fair point that, in ignoring cell turnover and other complexities in the infection process, we are potentially underestimating the amount of noise present in our (stochastic) spatial model for the viral dynamics, which may influence the strength of our inferences. In our original manuscript, we had avoided imposing artificial noise on observational data from the spatial model since the model is inherently stochastic. Moreover, we somewhat explored the role of added noise in the model in Results "Inference on the prevalence of cell–to–cell infection is robust to smaller samples of the cell sheet" by computing the clustering metric on smaller subsets of the cell sheet. Nonetheless, we agree with the reviewer that it is worthwhile to investigate specifically how an increase in observational noise would impact our results, as we already carried out for the ODE model (Supplementary Section "ODE model under varying observational noise"). We carried out this analysis in a new supplementary section and found that the parameter estimation with the spatial model was generally robust up to a substantial addition of observational noise. We made the following changes to include these results:

- **New Supplementary Section "Spatial model under varying (artificial) observational noise"**
  Refer to revised Supplementary Information

- Line 257 (revised manuscript): Added text in Results.

  The observational model used in our analysis here aims to recreate the noise incurred in an experimental setting. As such, we obtain our observational time series data by sampling one observation from each of a set of independent stochastic runs of the spatial model. This reflects the destruction of the cell culture in the observation process. However, it is certain that in experimental settings the observational process will incur additional noise than we have explicitly accounted for in this model. As such, we repeated the fitting process shown here after applying an additional negative binomial observational noise layer (the same as used for the ODE model) to both the fluorescence and clustering data. We discuss our results in Supplementary Section S1 and Supplementary Figure 2. Supplementary Figure 2 shows that estimates for both P_{CC} and t_{peak} with the spatial model are robust to a substantial degree of observational noise, especially when compared to applying the same levels of noise to the data and performing inference under the ODE model (shown in Supplementary Section S2). This finding suggests that estimates of P_{CC} using this approach is resilient to additional noise which may be incurred in an experimental setting.

Comment 2.3

> *In this context, the current title of the manuscript also might be a bit too strong/misleading. The authors provide a proof of concept based on simulated data, but an application to actual experimental data would be helpful to show its applicability (or what kind of estimates it would provide in comparison to other approaches cited, that generally predict*

a high proportion of cell-to-cell transmission in different viral infections, as cited by the authors (e.g. page 1 bottom, page 2 top)).

We agree with the reviewer that the original frontmatter of the manuscript was a little misleading due since we did not make it sufficiently clear that our paper is a simulation–estimation study. We felt that adding this fact directly into the title of the manuscript made the title unwieldy, however, we have revised the abstract and keywords of the manuscript to make it clear that our study is based on simulated and not experimental data.

- Adjusted Abstract and Keywords, additions in red:

  Abstract: The role of direct cell–to–cell spread in viral infections — where virions spread between host and susceptible cells without needing to be secreted into the extracellular environment — has come to be understood as essential to the dynamics of medically significant viruses like hepatitis C and influenza. Recent work in both the experimental and mathematical modelling literature has attempted to quantify the prevalence of cell–to–cell infection compared to the conventional free virus route using a variety of methods and experimental data. However, estimates are subject to significant uncertainty and moreover rely on data collected by inhibiting one mode of infection by either chemical or physical factors, which may influence the other mode of infection to an extent which is difficult to quantify. In this work, we conduct a simulation–estimation study to probe the practical identifiability of the proportion of cell–to–cell infection, using two standard mathematical models and synthetic data that would likely be realistic to obtain in the laboratory. We show that this quantity cannot be estimated using non–spatial data alone, and that the collection of a data which describes the spatial structure of the infection is necessary to infer the proportion of cell–to–cell infection. Our results provide guidance for the design of relevant experiments and mathematical tools for accurately inferring the prevalence of cell–to–cell infection in *in vitro* and *in vivo* contexts.

  Keywords: Cell–to–cell viral infection, simulation–estimation, viral dynamics, Bayesian inference, multicellular model.

- Line 409 (revised manuscript): Adjusted text in Discussion, additions in red:

  In this work we have conducted a number of simulation experiments to investigate the use of mathematical models in inferring the relative proportions of cell–to–cell and cell–free viral infection, which we summarised via the metric P_{CC}: the proportion of infections arising from the cell–to–cell route. We have applied simulation–estimation techniques using Bayesian methods for inference on both an ODE model and a spatially–explicit multicellular model. As much as possible, we aimed to emulate the type and quality of data available experimentally.

We certainly agree with the reviewer that an application to experimental data would help to demonstrate the value of our methods here; this is our first priority for future study. However, since the clustering metric, $\kappa(t)$, has not been collected (to our knowledge) in any experimental work to date, our method would require post–processing of fluorescence images of infected tissues at different points in time. We are not aware of any such images of sufficient size being available in the experimental literature, with the exception of large cell–culture images — particularly those of entire cell wells for plaque assays — which are too large to be processed manually and would require automated image analysis. In order to provide a thorough application of our method to experimental data, we would therefore need to substantially expand our methodology to include automated image processing, if not also to collect novel experimental data. This is beyond the scope of what we aim to achieve with the current manuscript, but is being actively investigated for future work. We have adjusted the discussion section of our manuscript to more clearly explain the extensions necessary to apply our approach to experimental data and why this is beyond the scope of our current study:

- Line 549 (revised manuscript): Adjusted text in Discussion, additions in red:

  Our hope is that this work provides the foundations for applying mathematical modelling and inference methods to real experimental data in order to accurately quantify the relative roles of cell–free and cell–to–cell spread in real viral infections. The obvious extension to our work here is to apply our methods to experimental data. The data sources we have assumed here — the fluorescent cell proportion time series and the time series for the clustering metric $\kappa(t)$ — are readily obtainable (or at least estimable) from model cellular systems. This could be achieved *in vitro* by following standard laboratory methods, and would only require simple staining and imaging techniques [38, 28, 21]. After harvesting and fixing the cell sheet at one of a specified set of observation times, fluorescent cells are easily identified by staining with fluorescent antibodies and imaging the cell sheet. The resulting image could then be processed to compute the fluorescent proportion of the cell population, and to compute or estimate the clustering metric $\kappa(t)$. We do not conduct such an analysis here, preferring instead to leave this for detailed study in a future work. In their study, Kongsomros and colleagues show images only of very small sections of the cell sheet consisting of approximately 10–15 cells, which is insufficient for inference [21]. By contrast, other available experimental images contain very large populations of cells which require automated image processing [37, 10]. Another potential obstacle to analysis of experimental data is in collecting data at a sufficient number of time points. Since time series data of the type assumed here involves destroying the cell sheet at the point of collection, it is expensive to collect data at fine time resolution [21, 28, 10]. We moreover explored the possible influence of additional observational noise that may be present in experimental data in Supplementary Section S2 and found that while additional observational noise reduces certainty in predictions of cell–to–cell infection proportions, it does not create systemic biases. These complications influencing the experimental application of our methods here will be explored in future studies.

Comment 2.4

> *The parameter inference step, as e.g. shown in Figure 2a-c for the three different parameter combinations and explained within the text, is partly difficult to follow. It is not totally clear how the different estimates (dots) of the different chains were obtained. In addition, I think it would be helpful to show a heat map of the estimates as shown in Figure 4a-c to infer the core area of the distributions. While the inclusion of the proposed clustering metric clearly improves the identification of the targeted quantity P_{CC} with reduced variation, it is not clear how the identification of the parameters alpha and beta might not be equally affected, given that Figure 2a-c and Figure 4a-c also show the parameters on two different scales. Besides inferring P_{CC}, it would also be interesting from a quantitative point of view to characterise these two parameters.*

The reviewer makes a good point that our original description of how the samples from the parameter inference for the ODE model was unclear in the text. We have revised the relevant section of text to clarify this. We also agree with the reviewer that a heat map — as we used for the spatial model in Figure 4(a)–(c) — is a better mode of communicating the shape of the posterior density than the scatter plot we used in the original version of Figure 2(a)–(c). We moved the existing scatter plot to the Supplementary Information as it shows that the different chains of the simulation–estimation mix well, and replaced the scatter plots in the main manuscript with heat maps in the same style as Figure 4(a)–(c). We provide a full list of changes below:

- Line 127 (revised manuscript): Adjusted text in Results, additions in red.

  Having obtained our observed data $\mathcal{D}$, we run a No U-Turn Sampling (NUTS) Markov Chain Monte Carlo (MCMC) algorithm [33] to obtain posterior density estimates for α and β. For each (α, β) pair to estimate, we run ten replicates of the simulation–estimation process. That

is, for each replicate we apply random observational noise to the true fluorescence data and then re-estimate α and β using four independent and randomly seeded chains. We draw 2000 samples from each chain and discard the first 200 samples as a burn–in. We assume uniform priors for α and β on $[0, 2.5]\,\mathrm{h}^{-1}$ and $\left[0, 2 \times 10^{-6}\right]\,(\mathrm{TCID}_{50}/\mathrm{ml})^{-1}\mathrm{h}^{-1}$ respectively, and assume a negative binomial likelihood. Further details of this simulation–estimation process are specified in Methods "Simulation–estimation".

In Figure 2, we show the results of this fitting process. In Figures 2a–2c, we show heat maps of the density of posterior samples in (α, β) space from each chain of a single replicate fit. We do so for each of the three target parameter pairs. As a visual aid, we also plot the (α, β) contours corresponding to the true P_{CC} value and the true r value in each case. These plots show that the posterior samples for each pair of target parameters are spread out along the true r contour. While some samples are close to the target parameter pair, the chains do not appear to converge at this point. In Supplementary Figure 3, we show an equivalent plot to Figures 2a–2c as a scatter plot of accepted samples, which confirms that the chains are indeed well-mixed.

- Changed Figure 2(a)–(c)

  Refer to revised manuscript

- Caption to Figure 2 (revised manuscript): Added text.

  (a)–(c) Posterior density as a contour plot in α–β space for a fit to fluorescence data with the ODE model where the true $P_{\mathrm{CC}} \approx 0.1, 0.5, 0.9$ and the value of r is held fixed. Density is shown for the 1800 samples from each chain after burn–in for a single replicate. We only show densities above a threshold value of 10^{-4}.

- **New Supplementary Section "Scatter plots for accepted posterior samples for the ODE model"**

  Contains posterior scatter plots from the original version of Figure 2(a)–(c).

The reviewer also raises a valid concern that we have not shown the distributions of α and β individually. This decision was initially made since we felt that these distributions did not especially add to the overall argument of the paper, in the sense that when P_{CC} was not well identified, neither were α and β, and when P_{CC} was precisely identified, so too were α and β. Nonetheless, this is understandably a point of interest for many readers and as such we have included the prior and posterior distributions for α and β for both the ODE model and the spatial model (with the clustering metric) in new Supplementary Sections.

- **New Supplementary Section "α and β marginal posterior distributions – ODE model"**
  Refer to revised Supplementary Information

- **New Supplementary Section "α and β marginal posterior distributions – spatial model with clustering data"**
  Refer to revised Supplementary Information

- Line 150 (revised manuscript): Added text in Results.

  The individual posterior distributions for α and β, which we show in Supplementary Figure 4, show a similar practical unidentifiability. While the mode of the distributions roughly follows the true values of these parameters, the sample densities are dispersed widely, hence confidence intervals on α and β are wide.

- Line 243 (revised manuscript): Added text in Results.

We also show the individual posterior distributions for α and β in Supplementary Figure 5. Supplementary Figure 5 shows a sharp peak of probability density around the true value of both α and β for each value of P_{CC}, especially when that mode of infection is minimal.

- Caption to Figure 2 (revised manuscript): Added text.

  The marginal posterior densities of α and β are shown in Supplementary Figure 4.

- Caption to Figure 4 (revised manuscript): Added text.

  The marginal posterior densities of α and β are shown in Supplementary Figure 5.

Comment 2.5

Page 2, second paragraph - There have been additional studies analysing cell-to-cell spread without this strict assumption and in complex environments (e.g. Imle et al. Nat Com 2019).

We thank the reviewer for pointing out this important omission from our survey of the literature. We have conducted a more thorough review of the literature on cell–to–cell and cell–free infection dynamics (also including the studies mentioned by the editor) and substantially revised our Introduction and Discussion sections. Please refer to our listed changes in response to the Editor's comments.

Comment 2.6

Page 13/Table 1: As the parameters used for simulation are very precisely indicated in Table 1 (i.e. several digits) and obtained from a fit of a model against experimental data, it seems a a bit irritating to not show the results ("for sake of brevity"). Although one could follow the argument that the actual values are not important, it is not clear if these really indicate realistic values. A comparison to other estimated rates (in this case for influenza infection) should be considered.

The reviewer makes a good point that the precision of our default parameter values requires further explanation. We had originally omitted discussion of how these parameters were estimated since we felt it distracted from the main objective of the manuscript and, as the reviewer points out, the exact values of these parameters are not especially important for our argument. However, in the interest of transparency and completeness, we agree that it is important to include our discussion of how these parameters were obtained. We have added a new Section "Parameter estimation for the ODE model" to the Supplementary Information which outlines our parameter estimation and shows a posterior predictive check which confirms that our default parameters are indeed reasonable. We have also modified the Methods text to refer to this section, as we outline below:

- **New Supplementary Section "Parameter estimation for the ODE model"**
  Refer to revised Supplementary Information

- Line 607 (revised manuscript): Adjusted text in Methods, additions in red.

  Throughout this work, we will assume fixed values of the parameters K, γ, δ, p, and c, as specified in Table 1. These parameters were obtained by running a Bayesian parameter estimation for the form of the ODE model as defined above against fluorescent cell time series data in Kongsomros *et al.* [21], and selecting one particular posterior sample at random. We sketch this parameter estimation process in Supplementary Section S12. These values were selected simply to be indicative of the realistic range of values for these parameters and are sufficiently realistic for the purposes of this work.

Reviewer 3 comments

Williams et al. used mathematical models to identify data types needed for inferring the fraction of cell-to-cell viral infection. The authors first showed that when only fluorescence time series data (representing the level of total infected cells) alone is available, inferring the fraction of cell-to-cell spread is impossible due to the inherent practical (un)identifiability issue with estimating cell-free infection and cell-to-cell infection independently. However, when another type of data, i.e. the proportion of infected neighboring cells over time, is available in addition to the fluorescence time series, inference of the fraction of cell-to-cell spread becomes feasible. The authors further constructed a spatial stochastic model of viral infection and used a Bayesian inference framework to demonstrate the accurate inference of the fraction of cell-to-cell spread using simulated data. Furthermore, the authors conducted sensitivity analysis to show that the inference is reasonably robust even when a small fraction of total cells are measured in the experiment.

The work is well written. Below, I list a few major concerns and some questions that need to be addressed.

Comment 3.1

The authors address an important question: whether a particular parameter of interest (in this case, the fraction of cell-to-cell spread) can be inferred from experimental data? Although the authors mentioned the work is motivated by data collected from Ref. 13, there is no description of the experimental data and the work is on using model inference on hypothetical and simulated datasets. Therefore, a major disconnect is how the framework developed here can be applied to real datasets. Adding more description of the type of datasets that are collected in the lab, discussion of the limitations associated with it (e.g. how noisy a typical data set is, whether time course data is possible for a single experiment, typically what fraction of cells are sampled etc.) are important to put this work into context. I assume that the real dataset is likely different from the simulated datasets in many aspects (e.g. noise level, and the number of time points). Using the framework to perform inference on real datasets (e.g. from Ref. 13) will be also important to demonstrate its utility in dealing with actual data.

The reviewer highlights an important disconnect between our work and the experimental literature. As we wrote in response to Comment 2.3 by Reviewer 2, a direct application of our analysis to experimental data is beyond the scope of this manuscript without a substantial expansion of methodology. This would be an investigation deserving of its own separate study, and one which we intend to carry out in future. However, the reviewer does make a good point that further discussion is needed in our manuscript to better explain the experimental data collection process and the quality and type of data available. To address this, and to clarify why an experimental application is beyond the scope of this study, we have revised the Discussion section of our manuscript as outlined below:

- Line 549 (revised manuscript): Adjusted text in Discussion, additions in red:

  Our hope is that this work provides the foundations for applying mathematical modelling and inference methods to real experimental data in order to accurately quantify the relative roles of cell–free and cell–to–cell spread in real viral infections. The obvious extension to our work here is to apply our methods to experimental data. The data sources we have assumed here — the fluorescent cell proportion time series and the time series for the clustering metric $\kappa(t)$ — are readily obtainable (or at least estimable) from model cellular systems. This could be achieved *in vitro* by following standard laboratory methods, and would only require simple staining and imaging techniques [38, 28, 21]. After harvesting and fixing the cell sheet at one of a specified set of observation times, fluorescent cells are

easily identified by staining with fluorescent antibodies and imaging the cell sheet. The resulting image could then be processed to compute the fluorescent proportion of the cell population, and to compute or estimate the clustering metric $\kappa(t)$. We do not conduct such an analysis here, preferring instead to leave this for detailed study in a future work. In their study, Kongsomros and colleagues show images only of very small sections of the cell sheet consisting of approximately 10–15 cells, which is insufficient for inference [21]. By contrast, other available experimental images contain very large populations of cells which require automated image processing [37, 10]. Another potential obstacle to analysis of experimental data is in collecting data at a sufficient number of time points. Since time series data of the type assumed here involves destroying the cell sheet at the point of collection, it is expensive to collect data at fine time resolution [21, 28, 10]. We moreover explored the possible influence of additional observational noise that may be present in experimental data in Supplementary Section S1 and found that while additional observational noise reduces certainty in predictions of cell–to–cell infection proportions, it does not create systemic biases. These complications influencing the experimental application of our methods here will be explored in future studies.

With regard to the reviewer's important query regarding the quality and quantity of available of experimental data available, we note that our choice of three-hourly collection points for obser-vatational data was an arbitrary one, but one which is within reason for laboratory experiments (Kongsomros et al. [21], for instance, collect data at 1, 3, 6, 12 and 24 hours post infection). Our model is inherently stochastic, and moreover by computing metrics on only samples of the tissue (Results "Inference on the prevalence of cell–to–cell infection is robust to smaller samples of the cell sheet") we introduce further noise to the data collection process, which we showed did not qualitatively worsen the quality of the inference. We have also explored more directly the role of observational noise by investigating the influence of an additional layer of noise on the quality of inference using the spatial model. We have added a Supplementary Section to include these results, which show that inference using the spatial model is robust for even high levels of (artificial) observational noise.

- **New Supplementary Section "Spatial model under varying (artificial) observational noise"**
  Refer to revised Supplementary Information

Comment 3.2

> *A key assumption in the model is that the cell-free spread is homogeneous, i.e. every cell is equally likely to be infected by a virus. While I agree this assumption is reasonable for other purposes, it may or may not be a good assumption for the inference here. In essence, the inference is to distinguish non-spatial pattern from spatial pattern of spread. This assumption of homogenous spread of cell-free infection depends on the size of the tissue being sampled and the diffusion of the virus relative to the size of the tissue. When the diffusion of the virus is low (in certain medium or in vivo), one will expect cell-free infection leads to infection of cells close by (as well as neighboring cells). This has been suggested for influenza infection in the upper respiratory tract and HCV infection in the liver. In this case, cell-free infection may look like cell-to-cell infection. I think it is important to discuss this complication and that the inference is not inferring cell-to-cell infection per se; rather it's an inference of spatial vs. non-spatial spread. Therefore, the inference may represent an upper bound for the fraction of true cell-to-cell spread. Also, I think this consideration may become important especially when only a fraction of cells are sampled in the experiment – in this case, one likely samples cells close by (rather than neighboring cells). Can the authors extend the spatial model to test how sensitive the inference to the homogenous assumption by assuming cell-to-cell spread follows diffusion pattern (where the diffusion constant is in a biologically plausible range), especially when a fraction of cells are sampled?*

The reviewer makes the same (very important) point as in Comment 2.1 by Reviewer 2. We refer to the substantial revisions made in response to Comment 2.1.

Reviewer 3 also mentions that the inclusion of additional noise from sampling only a fraction of the cell sheet — which we analysed separately in our original manuscript — may compound the challenge of determining the proportion of cell–to–cell infection when the viral density is not uniform. However, since when we sample cells we still examine the cell's true neighbours to determine the clustering metric. We therefore anticipate that while sampling a fraction of the cells introduces additional observational noise, it should not impose a spatial bias on the clustering metric. As such, we expect the sampling noise to not impose a systematic bias in our estimates. We believe our description of this cell sampling process was not sufficiently clear, which the reviewer also points out in the following comment, and which we have amended in our revised manuscript.

Comment 3.3

> *It is not clear how the clustering metric $k_i(t)$ is calculated in the scenario where only a fraction of cells are sampled. I assume the fraction is calculated by counting the sampled cells only in both numerator and denominator – this is not written in the manuscript. In this case, what qualifies a neighboring cell? I imagine when the fraction of sampled cells is low, none (or only few) of the sampled cells are 'actually' neighboring to each other? I think it is also important to discuss why the inference is still robust when only 50 or 100 cells out of 2500 cells are sampled (for whatever the definition of neighboring cell it is). Again, this question also points to the importance of testing the robustness of the inference when cell-free infection is not homogenous (as assumed).*

The reviewer makes a fair comment in pointing out that we have not sufficiently clearly defined the clustering metric where we only sample a fraction of the cells. In particular, we appear to have given the misleading impression that the clustering metric is computed by redefining cell adjacency on the sampled set of cells. In fact, we compute the clustering metric at each cell in the same way as with the entire sheet, by looking at each sampled cell's actual neighbours, which may or may not also be in the sampled set of cells. This means that, perhaps confusingly, we end up looking at more cells than are in the sampled set. We have clarified this computation in the relevant methods section and outline our changes below:

- Line 742 (revised manuscript): Adjusted text in Methods, additions in red.

  Given a cell grid where we denote by $\mathcal{F}(t)$ the set of cells which are fluorescent, we compute for each fluorescent cell $i \in \mathcal{F}(t)$ the quantity $k_i(t)$, which is the proportion of the neighbours of cell i which are also fluorescent. We then define $\kappa(t)$ as the mean of the $k_i(t)$s. We have:

  $$k_i(t) = \sum_{j \in \nu(i)} \frac{\mathbb{1}_{\{j \in \mathcal{F}(t)\}}}{|\nu(i)|} \tag{1}$$

  and

  $$\kappa(t) = \frac{1}{N} \sum_{i=1}^{N} k_i(t). \tag{2}$$

  We compute $\kappa(t)$ over time t to form a time series. In Figures 3a and 3g, we show an example of computing fluorescent neighbour proportions, and plot example $\kappa(t)$ time series for three parameter pairs, corresponding to P_{CC} values of 0.1, 0.5, and 0.9. Figure 3g shows that, unlike with the fluorescent cell time series, there is substantial variation in the $\kappa(t)$ curves with changing P_{CC}.

$\kappa(t)$ has the property that when it is near zero, fluorescent cells are mostly isolated and the infection is very diffuse, and when it is near one, fluorescent cells are generally found in clusters, indicating that the infection is very compact. In principle, $\kappa(t)$ could be computed or estimated in experimental settings with the use of fluorescence imaging of the cell sheet, samples of which can be found in works by Kongsomros *et al.* and Fukuyama *et al.* [21, 10].

We modify the definition of $\kappa(t)$ to define the approximation $\kappa_S(t)$ as follows. Given a grid of N cells at time t, of which the fluorescent population is given by $\mathcal{F}(t)$ as before, we draw $S \leq N$ cells without replacement and call the set of sampled cells $\mathcal{S}(t)$. For each sampled cell i, if $i \in \mathcal{F}(t)$, we compute $k_i(t)$, and then compute the approximate clustering metric $\kappa_S(t)$ as the mean of the computed $k_i(t)$s, that is, if $|\mathcal{S}(t) \cap \mathcal{F}(t)| \neq 0$, then

$$\kappa_S(t) = \sum_{i \in \mathcal{S}(t) \cap \mathcal{F}(t)} \frac{k_i(t)}{|\mathcal{S}(t) \cap \mathcal{F}(t)|}.$$

Note that $k_i(t)$ is defined as above. That is, for each sampled cell i, we still compute $k_i(t)$ from that cell's neighbours, which may not be in the sampled set $\mathcal{S}(t)$. In the event that no fluorescent cells are sampled (that is, $|\mathcal{S}(t) \cap \mathcal{F}(t)| = 0$), we define $\kappa_S(t) = 0$.

Comment 3.4

> *(A minor point) I believe the framework presented here only applies to viral infections where the target cells do not move. It will not work for infections such as HIV (where target cells move constantly). I think the scope of the framework should be mentioned throughout the manuscript.*

This is an important nuance, given that the literature on distinguishing the modes of viral spread is most developed for HIV, which we did not make sufficiently clear in our original manuscript. To address this, we have altered our introduction and discussion text to clarify the limitations of our study.

- Line 70 (revised manuscript): Added text in Introduction, additions in red:

  It is important to mention that the analysis which we conduct here is limited to infections of static tissues, and does not extent to infections in motile cell populations, such as HIV. Since, in this case, the migration of target cells enables well–mixed conditions, there is no notable spatial structure to infection and thus the resulting dynamics are less easily distinguished.

- Line 492 (revised manuscript): Added text in Discussion, additions in red:

  It is important to clarify here that our use of a global extracellular viral population is based on an assumption of rapid viral transport. This is an important distinction from the modelling literature on HIV (e.g. [17, 14, 20, 18]), where the system can be characterised by well–mixed dynamics since the target cells are also motile. This fact substantially changes the mode of action of the cell–to–cell mechanism and thus also the spatial structure of the infection. For this reason, the methods we have developed here do not extend to HIV infection.
